# Tumor Cell Marker PVRL4 (Nectin 4) Is an Epithelial Cell Receptor for Measles Virus

**DOI:** 10.1371/journal.ppat.1002240

**Published:** 2011-08-25

**Authors:** Ryan S. Noyce, Daniel G. Bondre, Michael N. Ha, Liang-Tzung Lin, Gary Sisson, Ming-Sound Tsao, Christopher D. Richardson

**Affiliations:** 1 Department of Microbiology and Immunology, Dalhousie University, Halifax, Nova Scotia, Canada; 2 IWK Health Sciences Centre, Canadian Center for Vaccinology, Halifax, Nova Scotia, Canada; 3 Department of Medical Biophysics, University of Toronto, Toronto, Canada; 4 Ontario Cancer Institute and Princess Margaret Hospital, Toronto, Canada; 5 Department of Pediatrics, Dalhousie University, Halifax, Nova Scotia, Canada; The Fox Chase Cancer Center, United States of America

## Abstract

Vaccine and laboratory adapted strains of measles virus can use CD46 as a receptor to infect many human cell lines. However, wild type isolates of measles virus cannot use CD46, and they infect activated lymphocytes, dendritic cells, and macrophages via the receptor CD150/SLAM. Wild type virus can also infect epithelial cells of the respiratory tract through an unidentified receptor. We demonstrate that wild type measles virus infects primary airway epithelial cells grown in fetal calf serum and many adenocarcinoma cell lines of the lung, breast, and colon. Transfection of non-infectable adenocarcinoma cell lines with an expression vector encoding CD150/SLAM rendered them susceptible to measles virus, indicating that they were virus replication competent, but lacked a receptor for virus attachment and entry. Microarray analysis of susceptible versus non-susceptible cell lines was performed, and comparison of membrane protein gene transcripts produced a list of 11 candidate receptors. Of these, only the human tumor cell marker PVRL4 (Nectin 4) rendered cells amenable to measles virus infections. Flow cytometry confirmed that PVRL4 is highly expressed on the surfaces of susceptible lung, breast, and colon adenocarcinoma cell lines. Measles virus preferentially infected adenocarcinoma cell lines from the apical surface, although basolateral infection was observed with reduced kinetics. Confocal immune fluorescence microscopy and surface biotinylation experiments revealed that PVRL4 was expressed on both the apical and basolateral surfaces of these cell lines. Antibodies and siRNA directed against PVRL4 were able to block measles virus infections in MCF7 and NCI-H358 cancer cells. A virus binding assay indicated that PVRL4 was a *bona fide* receptor that supported virus attachment to the host cell. Several strains of measles virus were also shown to use PVRL4 as a receptor. Measles virus infection reduced PVRL4 surface expression in MCF7 cells, a property that is characteristic of receptor-associated viral infections.

## Author Summary

Measles virus is a primate-specific virus that causes acute respiratory disease and can also lead to short term immune suppression resulting in secondary infections by bacteria or parasites. Wild type measles virus attaches to and infects lymphocytes using the receptor CD150 (signaling lymphocyte activation molecule, SLAM). Measles virus is also known to infect epithelial cells of the upper respiratory system and lungs. However, the viral receptor on these cells was previously unknown. Adenocarcinomas are derived from glandular epithelial cells of organs including the lung, breast, or colon. We showed that wild type isolates of measles virus can infect human airway epithelial cells and many adenocarcinoma cell lines. A comparative analysis of membrane genes expressed in cells susceptible and non-susceptible for measles virus infections revealed candidate receptor proteins. Only PVRL4 (Nectin 4) converted cells that were resistant to measles viral infections, to cells that could support virus infections. PVRL4 is a tumor cell marker that is highly expressed on embryonic cells such as those of the placenta, but it is also expressed at lower levels in the trachea, oral mucosa, nasopharynx, and lungs. It is highly expressed on many lung, breast, colon, and ovarian tumors suggesting that they could be targeted with oncolytic measles virus.

## Introduction

In spite of the success of an attenuated measles virus (MV) vaccine in the modern world [1] measles virus (MV) is still a major killer of children in developing countries [2]. MV strikes an estimated 20 million children a year and killed around 164,000 individuals in 2008 according to the World Health Organization (http://www.who.int/mediacentre/factsheets/fs286/en/). MV causes an acute disease characterized by fever, photophobia, coughing, running nose, nausea, and a macular red rash over most of the body. In rare instances, persistent MV infections can occur in the brain and lead to encephalitis. Humans and monkeys are hosts for MV [3]-[7] while most rodents are not normally infected by the virus [8]–[10]. The recent discovery that attenuated strains of MV possess oncolytic properties and can be used to destroy tumor cells, has kindled an interest in this virus as a gene therapy agent [11], [12].

Measles virions contain a negative strand RNA genome from which viral mRNAs are transcribed to encode a nucleocapsid protein (NP), a phosphoprotein (P), virulence factors (C and V), matrix protein (M), membrane fusion protein (F), the hemagglutinin/receptor binding protein (H), and an RNA polymerase (L) [13]. Surrounding the nucleocapsid is a membrane which contains the two viral glycoproteins, H and F. The H protein is required for viral attachment to the host cell receptor, while F mediates membrane fusion and entry at the host plasma membrane and is also responsible for syncytia (multi-nucleated cell) formation.

Interaction of the H protein of MV with a cellular attachment factor is the initial event of infection. The binding of H to the host cell receptor triggers and activates the F protein to induce fusion between virus and host cell membranes [14]–[16]. The search for MV cellular receptors initially began with vaccine/laboratory strains and progressed to more relevant receptors used by wild type MV (wtMV) isolates [17]. Human membrane cofactor protein (MCP/CD46) is a receptor for the Edmonston laboratory/vaccine strain of MV [18], [19]. CD46 is a complement regulatory protein that is expressed on most cell types in the human body, with the exception of red blood cells (although it is on monkey erythrocytes) [20]. Natural isolates of wtMV can be adapted to grow in Vero monkey kidney cells and this is accompanied by mutations in the H protein that convey the CD46 receptor binding phenotype [21]–[23]. Strains of wtMV are routinely isolated in marmoset B95-8 cells, a B cell line immortalized with Epstein-Barr virus, which allows the virus to grow without the need for adaptation [24]. These isolates cannot use CD46 as a receptor [22], [25]. Our laboratory and others hypothesized that another lymphotropic receptor could be used by wild type isolates of MV [22], [26], [27]. Signaling lymphocyte activation molecule (SLAM) or CD150 was identified to be a lymphotropic receptor for both clinical isolates and vaccine strains of MV [28]–[30]. SLAM/CD150 is a signaling molecule that is expressed on activated B, T, monocyte, and dendritic cells [31].

Recent evidence indicates that CD150^+^ alveolar macrophages, dendritic cells, and lymphocytes are the initial targets for measles virus infections in macaques [32]–[35]. However, wild type MV, in autopsied human patients and some experimentally infected monkeys, has been shown to infect the epithelial cells of the trachea, bronchial tubes, lungs, oral cavity, pharynx, esophagus, intestines, liver, and bladder [36], [37]. These epithelial cells do not express SLAM/CD150, but the infected cells do shed virus [37]–[39]. Epithelial cells may be important later on in infection and for the spread of MV by aerosol droplets. Wild type MV does not readily infect most common laboratory epithelial, endothelial, or fibroblast cell lines. In addition, cryo-preserved primary human small airway epithelial cells (SAEC) grown in serum free epithelial cell growth medium are not normally susceptible to wtMV, but can be made susceptible by culturing them in 2% fetal calf serum [39]. These cells do not express CD150/SLAM and the wtMV cannot use CD46/MCP, suggesting that there is another receptor on epithelial cells [39]. The third receptor has been postulated to lie on the basolateral side of epithelial cells in close context to infected lymphocytes and dendritic cells, and it may play a secondary role following infection of the lymphatic system that is important for MV transmission [33], [34], [40]–[42]. Virus is postulated to infect epithelial cells using the unidentified basolateral receptor, and MV is subsequently shed from the apical surface of these cells [33], [42], [43]. Other investigators have been searching for an elusive receptor on polarized epithelial and cancer cell lines [41], [44]–[46]. Recently it was shown that loss of tight junction proteins, during an epithelial-mesenchymal cell transition induced by the transcription repressor SNAIL, blocked infections by wtMV [45]. It has become clear that tight junction proteins can serve as entry factors for other viruses that target polarized epithelial cells. These include hepatitis C virus, reovirus, herpes simplex virus and coxsackie virus [47]–[50]. The region of the H protein that interacts with the putative epithelial receptor was recently mapped on the 3-D structure of the viral attachment protein to include residues I456, L464, L482, P497, Y541, and Y543 [33], [41].

Here we show that wild type MV infects primary airway epithelial cells grown in fetal calf serum and many adenocarcinoma cell lines of the lung, breast, and colon. A microarray analysis of susceptible versus non-susceptible cell lines showed that transcripts for many adherens junction and tight junction proteins are up-regulated in virus receptive cells. However, the integrity of these junctions was not a prerequisite for infection. Non-susceptible cell lines could be infected following transfection with a CD150/SLAM expression vector, indicating that they were replication competent. Analysis of the microarray data, filtered for membrane protein genes, produced a short list of 11 candidate receptors. Of these only human PVRL4 (Nectin 4), a tumor cell marker found on breast, lung, and ovarian carcinomas, rendered cells amenable to MV infections. Transient knockdown of PVRL4 using siRNA abolished wtMV infection in these cell lines. The identification of this receptor could provide impetus for MV as an oncolytic treatment for lung, breast, and colon adenocarcinomas.

## Results

### Wild type MV infects serum activated SAEC independently of CD46 (MCP) and CD150 (SLAM)

**Figure 1:**
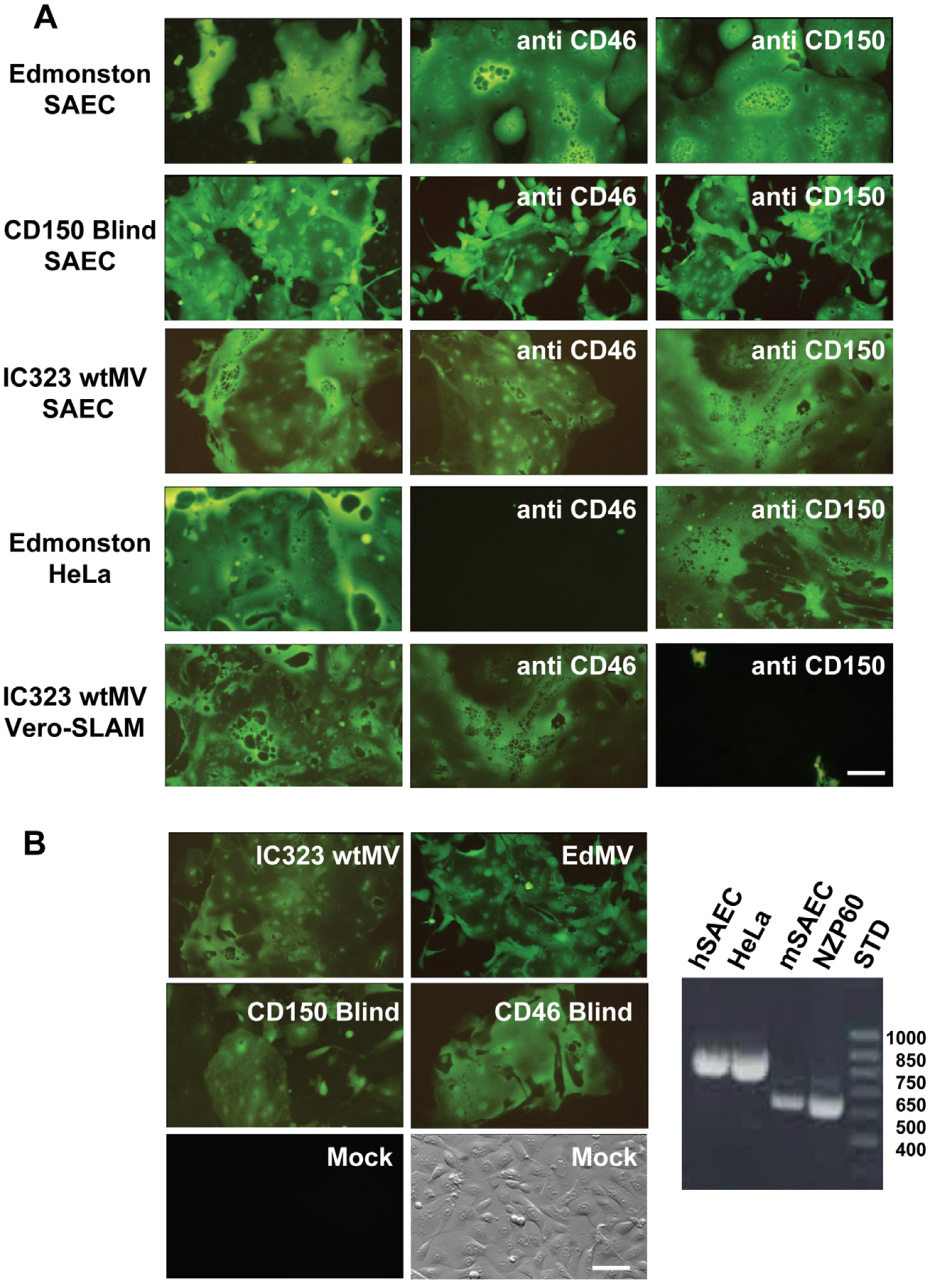
A new receptor for MV is present on smooth airway epithelial cells (SAEC). (A) Human SAEC were incubated with receptor neutralizing antibodies against CD46 (M75 and B97) or CD150 (IPO-3 and A12) and challenged with the Edmonston vaccine, CD150 Blind, or IC323 wild type strains of MV. Each virus strain contained the EGFP reporter gene. In virus control experiments antibodies against CD46 inhibited infection by Edmonston MV in HeLa cells while antibodies against CD150 blocked infection of Vero-CD150/SLAM by wild type IC323 MV. (B) Marmoset SAEC contain a deletion of the SCR1 domain of CD46 and do not express CD150/SLAM. The panel on the right shows a diagnostic PCR spanning the SCR1 domain revealed by agarose gel electorphoresis in the presence of ethidium bromide, that confirms the deletion in marmoset SAEC. However, the marmoset SAEC could be infected with either the Edmonston or IC323 strains of MV. Virus containing H protein that was mutated in either its CD150 binding site (CD150 Blind) or its CD46 binding site (CD46 Blind) also replicated in the marmoset SAEC. Scale bar = 100 µm.

Human primary SAEC were previously shown to support wtMV replication and produce syncytia when grown in the presence of 2% fetal calf serum but not in serum free media. These cells did not express CD150 (SLAM) [39]. We confirmed these results and further demonstrated that infections with a recombinant wtMV engineered to express EGFP (IC323-EGFP wtMV) were independent of CD46 (MCP) and CD150 (SLAM) expression. Infections with IC323-EGFP wtMV were unaffected by the presence of monoclonal antibodies directed against CD46 and CD150, that were previously shown to neutralize MV infections [44], [51] (Figure 1A). SLAM blind virus, which contains mutations in the H protein that prevents CD150 recognition, along with an EGFP reporter gene [52], also infected these cells. Marmoset cell lines do not express the critical SCR1 virus binding domain of CD46 [53], [54]. Deletion of SCR1 in the marmoset SAEC was confirmed by diagnostic RT-PCR of CD46 mRNA using conserved primer sequences (Figure 1B). However, marmoset SAEC were still susceptible to IC323-EGFP wtMV (Figure 1B). The marmoset cells could also be infected with Edmonston-EGFP, SLAM blind and CD46 blind recombinant MV [52] (Figure 1B). These results provide further support for the existence of a unique MV epithelial cell receptor.

### Wild type MV infects adenocarcinoma cells derived from lung, breast, and colon tumors

Since adenocarcinomas are defined as tumors which are derived from glandular epithelial cells, we decided to test the susceptibility of a number of different tumor cell lines to infection with IC323-EGFP wtMV. Infectivity assays were performed on 12 lung, 4 breast, 6 colon, 3 liver, 1 pancreatic, 1 cervix and 5 kidney cell lines. The relative infectivity in the different cell lines was assessed qualitatively, as the percentage of fluorescent cells due to virus-mediated EGFP expression (Table 1). Most adenocarcinomas were susceptible to IC323-EGFP MV infection, and the exceptions were A549 (lung), MDA-MB-231 (breast), HCT116 (colon), HepG2 (liver), HS766T (pancreas), and HeLa (cervix) cells, which were non-susceptible to the virus (Figure S1 in Text S1). Large cell and small cell carcinoma cell lines from the lung also did not support infection. To determine whether the non-susceptible property of negative cell lines was due to the absence of a particular receptor, non-susceptible cell lines were transfected with a cDNA expression plasmid for the lymphotropic receptor CD150/SLAM. Expression of CD150 rendered A549, MDA-MB-231, HeLa, Vero, and OMK cells susceptible to IC323-EGFP wtMV, indicating that the cells were competent for MV replication, but lacked the entry protein(s) for viral infection (Figure S2 in Text S1).

**Table 1:** Adenocarcinoma cell lines tested for susceptibility to wt MV-EGFP infection.

Tissue Type	Cell Line	Tumour Type	% Infection
			Efficiency
Lung	MGH24	adenocarcinoma	+++++
	NCI-H358	adenocarcinoma	+++++
	NCI-H125	adenocarcinoma	++++
	Calu-3	adenocarcinoma	++++
	RVH6847	adenocarcinoma	+
	A549	adenocarcinoma	-
	SBC-3	small cell carcinoma	-
	MGH7	squamous cell carcinoma	-
	NCI-H157	squamous cell carcinoma	-
	NCI-H460	large cell carcinoma	-
	NCI-H661	large cell carcinoma	-
	NCI-H520	squamous cell carcinoma	-
	NCI-H226	squamous cell carcinoma	-
Breast	MCF7	adenocarcinoma	+++++
	MDA-MB-468	adenocarcinoma	+++++
	T47D	adenocarcinoma	++++
	MDA-MB-231	adenocarcinoma	-
Colon	DLD-1	adenocarcinoma	+++++
	LoVo	adenocarcinoma	+++++
	T84	adenocarcinoma	++++
	HT29	adenocarcinoma	++++
	HCT116	adenocarcinoma	-
Liver	Huh7	adenocarcinoma	+
	Hep3B	adenocarcinoma	+
Pancreas	HS766T	adenocarcinoma	-
Cervix	HeLa	adenocarcinoma	-
Kidney	MDCK (dog)	n.a.	+/−
	Vero (green monkey)	n.a.	+/−
	HEK 293 (human)	n.a.	+/−
	COS-1 (green monkey)	n.a.	+/−
	OMK (owl monkey)	n.a.	-
	NZP60 (marmoset)	n.a.	-
	BHK21 (hamster)	n.a.	+/−
Ovary	CHO (hamster)	n.a.	-

See also Figure S1 in Text S1.

+++++ 100% cells infected; ++++ 80% cells infected; +++ 60% cells infected; ++ 40% cell infected; + 20% cells infected; +/− 5% cells infected; - 0% cells infected.

### Microarray analysis reveals that PVRL4 (Nectin 4) is a receptor for wtMV

**Figure 2:**
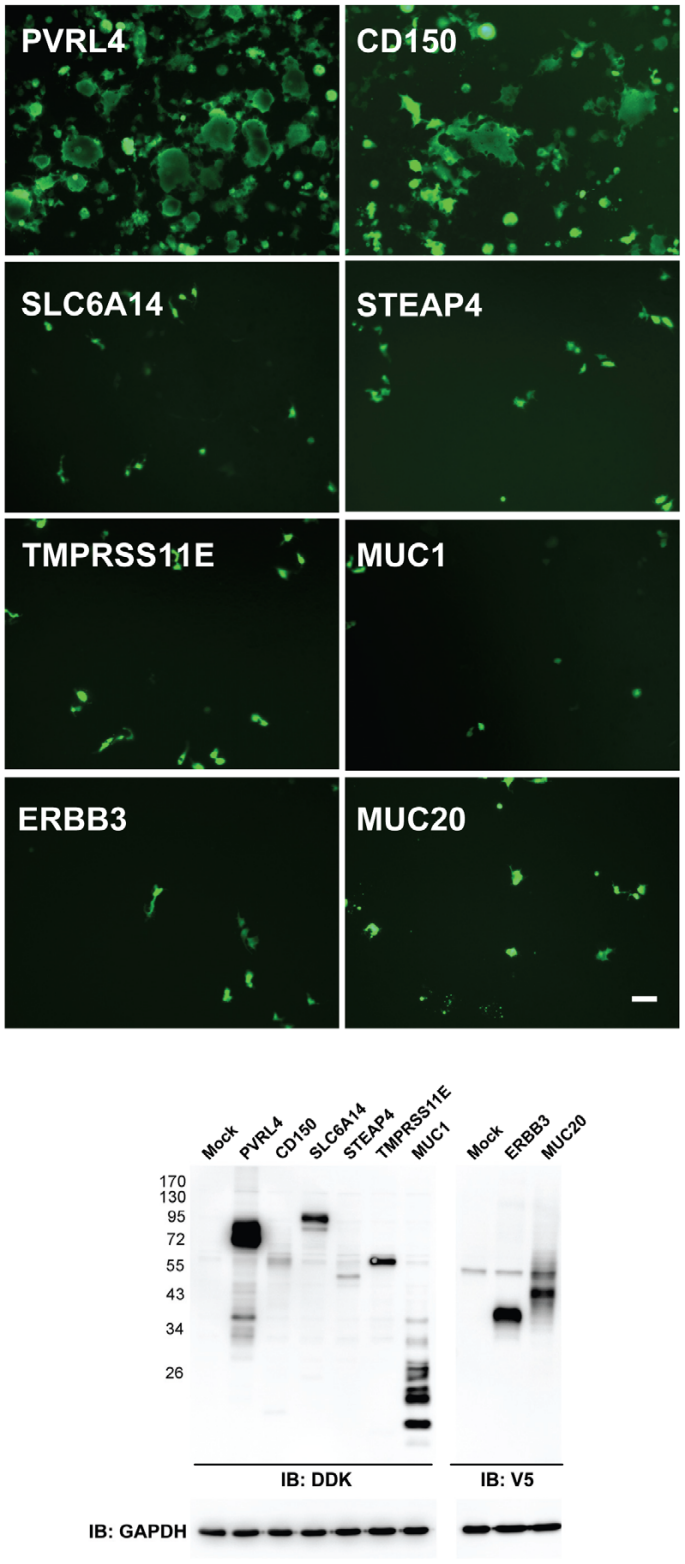
PVRL4 (Nectin 4) can function as an entry factor for IC323-EGFP wtMV. COS-1 cells were transfected with expression plasmids containing the coding sequences for candidate membrane protein receptors. After 36 hrs the cells were infected with IC323-EGFP wtMV. Virus specific fluorescence was observed between 24–48 hrs infection at 100x magnification using a Leica inverted microscope. Both PVRL4 (Nectin 4) and the positive control CD150/SLAM were capable of converting the non-susceptible COS-1 cells to a virus susceptible phenotype that produced syncytia. Other candidate receptor proteins including SLC6A14, STEAP4, TMPRSS11E, MUC1, ERBB3, and MUC20 were ineffective in producing infections, and yielded only isolated background single-cell infections that did not produce syncytia. Whole cell protein lysates were separated by SDS-PAGE followed by Western Immunoblot using Flag (IB: DDK) and V5 (IB: V5) antibodies to detect expression of these candidate receptors. GAPDH was used as a loading control. Scale bar = 100 µm. See also Figures S2 and S3 in Text S1.

Microarray analysis and a comparison between susceptible and non-susceptible cells were previously used to identify the cellular receptor for Nipah virus [55]. In our case the mRNA transcripts from cells that were susceptible to wtMV infection were compared to those from non-susceptible cells using the Affymetrix Human Gene ST 1.0 Array. RNA was prepared from breast adenocarcinoma (MCF7, MDA-MB-468, T47D, MDA-MB-231), lung adenocarinoma (NCI-H358, MGH24, NCI-H125, A549), and SAEC (with and without serum treatment) cell lines. Following the analysis it was apparent that many of the up-regulated membrane proteins were associated with the tight junctions and adherens junctions found in polarized epithelial cells (Table S1). Recently another laboratory reported that loss of tight junctions, during an epithelial-mesenchymal cell transition induced by the transcription repressor SNAIL, blocked receptor-dependent infections by wtMV [45]. The percentage up-regulation of gene expression for membrane proteins in susceptible cells compared to non-susceptible cells was calculated for breast, lung, and SAEC categories of cell lines (Table S1). These values were ordered and only gene products which were up-regulated greater than 20% were considered in our analysis. Evaluation of potential receptors was conducted in 2 phases. Gene products that were up-regulated in susceptible breast adenocarcinomas were first compared to those up-regulated in susceptible lung adenocarcinomas. To investigate whether this subset of candidate receptor genes from the initial microarray screens might act as an epithelial receptor for wtMV, we cloned these genes from a cDNA library of membrane proteins from Open Biosystems (Huntsville, AL) or purchased the genes not represented in this library from Origene Systems (Rockville, MD). We chose to introduce the expression plasmids into COS-1 monkey kidney cells due to their high transfection efficiency. Expression of the individual candidate receptor genes was verified by Western immunoblot analysis for the V5 peptide tag that was fused to the carboxy terminus of each membrane protein from the Open Biosystems vectors or the Myc-DDK(Flag) tag from the Origene vectors (Figure 2, Figure S3 in Text S1). At 36 hours post-transfection, COS-1 cells were inoculated with wtMV-EGFP and infections were monitored between 24-72 hours p.i. Over 48 membrane protein genes that were the most highly up-regulated in both breast and lung adenocarinoma cells were originally tested without success (indicated with * in Table 2). Subsequently, in the next phase of testing, the up-regulated genes common to both breast and lung adenocarcinomas were compared to those in serum activated SAEC cells. The results are presented in Table 2, and 11 common gene products were over-expressed in all 3 tissue types. These candidate receptor genes included SLC6A14, STEAP4, TMPRSS11E, MUC1, ERBB3, PVRL4, MUC15, PCDH1, ANO1, MUC20, and CLDN7. Of these, 10 were tested (indicated with ** in Table 2) and it became immediately evident that PVRL4 could act as a receptor and facilitate infection (Figure 2). (Both PVRL4 (Nectin 4) and the CD150/SLAM positive control yielded infections that were characterized by syncytia formation with typical MV cytopathology. A background of single infected COS-1 cells which did not fuse and form syncytia was also evident. Infections in these cells did not progress and could be due to another route of entry such as macropinocytosis. These single infected cells were previously reported in MV infected CHO and Vero monkey kidney cells and occurred at frequency of 2–3 logs below that of SLAM-dependent infections [44]. This background could not be eliminated with siRNAs directed against PVRL4 (data not shown). Expression of exogenous PVRL4 in other non-susceptible cell lines (OMK, HeLa, A549, and MDA-MB-231) also rendered them susceptible to IC323-EGFP wtMV infection (Figure S2 in Text S1).

**Table 2:** Gene products up-regulated in susceptible breast, lung, and SAEC cell lines compared to non-susceptible cells.

Common Genes Up-regulated in Breast & Lung Cells	% Gene Up- Regulation in Breast Cells	% Gene Up- Regulation in Lung Cells	Common Genes Up-regulated in Breast, Lung, SAEC	% Gene Up- Regulation in SAEC
SLC6A14*	173.1415	61.90872	SLC6A14**	46.87437
RAB25 *	161.964	188.6348		
CDH1*	131.1153	45.16778		
GPC4*	103.9329	129.3733		
STEAP4*	100.7787	109.9432	STEAP4**	64.64598
TMPRSS11E*	96.91898	118.1558	TMPRSS11E**	46.68319
NCAM2*	94.40557	43.07266		
CDH3*	93.77679	123.3686		
FXYD3	86.12615	41.92274		
MUC1*	75.34311	49.158	MUC1**	32.83022
MME	73.32612	82.94319		
ERBB3*	72.10978	45.78744	ERBB3**	23.24152
PCDHB8*	70.45194	89.7596		
ST14*	68.33217	106.8741		
GABRA3	65.42129	50.05689		
PRSS8	58.427	52.65615		
PCDHB4*	57.51795	53.33358		
SLC16A14*	55.81763	38.13178		
ANK3	51.61145	44.22223		
PVRL4	50.68993	38.80007	PVRL4**	27.6538
MUC15*	47.35872	60.32913	MUC15**	23.22254
SYK	47.19083	68.0141		
SCNN1A*	47.04117	64.89888		
PCDH1*	41.70316	41.02153	PCDH1**	22.85412
FAP	40.45849	38.08091		
OR8G5	40.37826	51.7274		
ANO1	38.69293	45.62884	ANO1	40.30204
MUC20*	37.97506	45.12805	MUC20**	44.45975
PROM2*	37.844	50.14194		
SUSD4*	37.46031	27.36689		
EPCAM*	37.39759	116.3044		
FGFBP1*	36.91295	38.48566		
EPHA1*	35.75165	50.02149		
EPCAM *	35.06523	95.96663		
ENPEP	34.85825	77.82285		
IGSF9	34.23295	35.2908		
CHRM3	32.84461	47.77368		
PCDHB15	30.85493	45.72636		
CLDN7*	29.9608	84.29241	CLDN7**	26.90155
RAB19	28.47934	39.00469		
DSC2	27.83151	45.32287		
MMP16	27.7552	27.27076		
PSD4	26.42839	37.93089		
MAL2*	25.88348	184.0902		
GJB5	25.58353	43.39558		
GPR81	25.39142	115.9613		
ADAP1	25.17025	43.49649		
VEPH1	24.12028	35.18223		
PCDHB13	23.84833	85.86242		

See also Table S1.

*Primary screening of candidate receptors with cDNA expression vectors following comparison of lung and breast cancer cell lines0.

**Secondary screening of candidate receptors with cDNA expression vectors following comparison of lung, breast, and SAEC cell lines.

### Related proteins (PVR, PVRL1, PVRL2, PVRL2) cannot function as a receptor for wtMV

**Figure 3:**
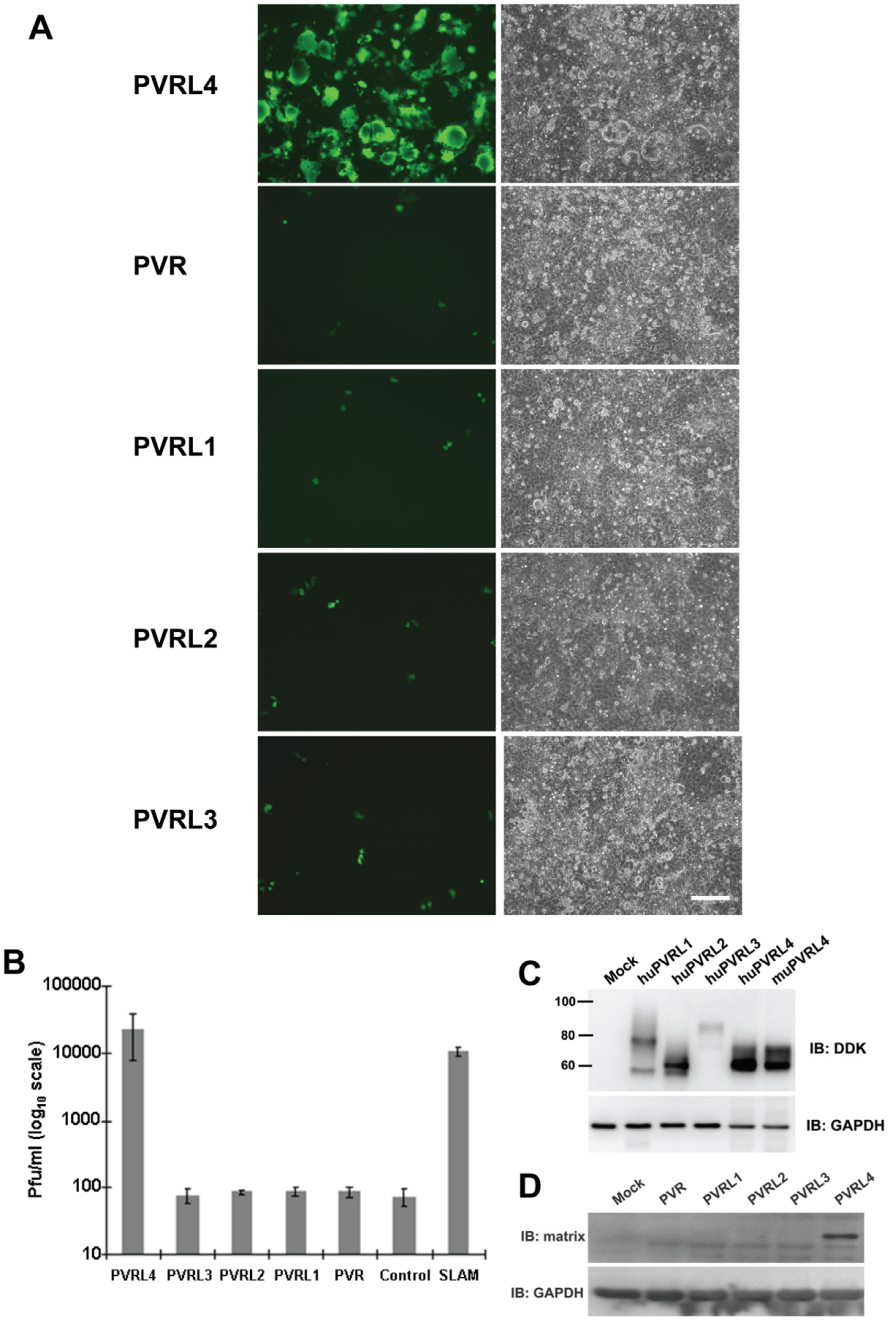
Nectins closely related to PVRL4 cannot function as receptors for wtMV. COS-1 cells were transfected with expression vectors encoding DDK-tagged versions of PVR, PVRL1, PVRL2, PVRL3, and PVRL4. Control cells were transfected with empty plasmid. After 36 hrs, the transfected cells were infected with IC323-EGFP wtMV and incubated a further 48 hrs. (A) Cells were viewed by fluorescence and phase contrast microscopy. Scale bar  = 200 µm. (B) Virus released from the infected cells was quantified by plaque assay. Data are expressed as the mean of three independent experiments, with error bars showing the SEM. (C) Total cell expression of the transfected proteins was evaluated by Western immunoblots using antibodies directed against the DDK(Flag). (D) Viral proteins were synthesized in PVRL4 transfected cells following MV infection as shown by Western immunoblot using an antibody specific for the viral matrix (M) protein.

PVR, PVRL1, PVRL2, and PVRL3 are nectin proteins that are closely related in structure and sequence to PVRL4 (Figure S4 in Text S1). The proteins PVR, PVRL1, and PVRL2 have previously been shown to function as receptors for polio (PVR) and herpes simplex (PVRL1, PVRL2) viruses. We tested the ability of PVR, PVRL1, PVRL2, and PVRL3 to function as receptors for MV following transfection into COS-1 cells. Fluorescence microscopy of non-permeabilized cells that over-expressed PVRL1, PVRL2, PVRL3, and PVRL4 confirmed cell surface expression of these proteins (data not shown). Only PVRL4 was capable of converting the non-susceptible cells to a wtMV susceptible phenotype (Figure 3A). Infected cells expressing PVRL4 produced virus particles based upon plaque assays (Figure 3B). Expression of the various nectin proteins was confirmed by SDS PAGE followed by immunoblot analysis using antibodies directed against the DDK tag (Figure 3C). Cells containing PVRL4 but not the other nectins also synthesized MV proteins as shown by an immunoblot for viral matrix (M) protein (Figure 3D).

### Susceptible but not non-susceptible cell lines express PVRL4 (Nectin 4) on their cell surface

**Figure 4:**
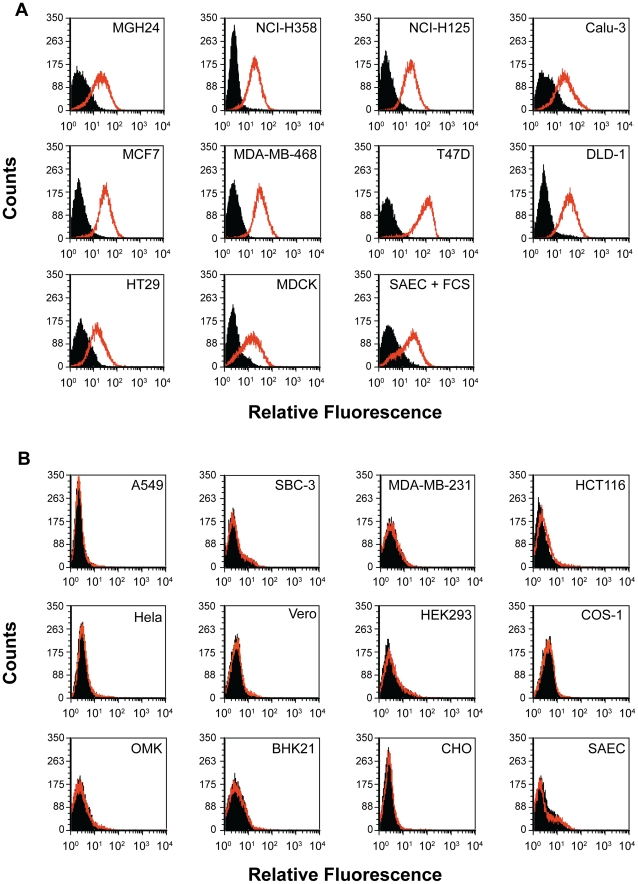
Flow cytometry analysis reveals PVRL4 (Nectin 4) surface expression on cells susceptible for wild type MV infections. (A) Susceptible cell lines were incubated with a phycoerythrin-conjugated mouse monoclonal antibody that was specific for human PVRL4 (red histogram) or a PE-conjugated mouse IgG2a control antibody (shaded histogram). Cells were washed and analyzed with a Beckman-Coulter ADP Cyan flow cytometer. The Y-axis represents cell counts and the X-axis represents fluorescence intensity. (B) Non-susceptible cell lines were analyzed as described for Panel A.

Flow cytometry was used to determine whether epithelial or adenocarcinoma cells that are susceptible for wtMV infection expressed PVRL4 on their surfaces. Cells susceptible for wtMV infection bound fluorescent antibodies specific for PVRL4 (Figure 4A). Non-susceptible cells, on the other hand, exhibited no difference in fluorescence when compared to the isotype control antibody (Figure 4B). NCI-H358, NCI-H125, MGH24, and Calu-3 lung adenocarcinoma cells expressed PVRL4 while A549 adenocarcinoma, squamous cell (NCI-H157), small cell (SBC-3), and large cell (NCI-H460) lung carcinomas did not. MCF7, MDA-MB-468, and T47D breast adenocarcinomas were PVRL4 positive, while the non-susceptible MDA-MB-231 cells were not. Of the colon tumor cell lines, HT29, T84, and DLD-1 cells were positive for PVRL4, while HCT116 cells were negative. Other adenocarcinomas of the liver (HepG2), cervix (HeLa), and kidney epithelial cell lines were negative for PVRL4 on their surfaces. Interestingly, SAEC treated with FCS for 24 h exhibited an increased level of PVRL4 on their surface (SAEC + FCS), whereas the SAEC cultured in the absence of serum did not. Since we and others have shown that SAEC grown in the presence of serum acquire the ability to become infected with wtMV, these data suggest that PVRL4 is an authentic epithelial cell receptor for MV. MDCK cells, which were originally derived from dog kidneys, also express PVRL4 on their surface. However, they are not susceptible to wtMV infections, suggesting that differences in the protein sequence of canine PVRL4 may reduce its ability to serve as a receptor for wtMV. In all epithelia derived cell lines that were tested, the presence of cell surface PVRL4 correlates with their ability to be infected with wtMV.

### The epithelial cell receptor is expressed on the apical and basolateral surfaces of polarized adenocarcinoma cells

**Figure 5:**
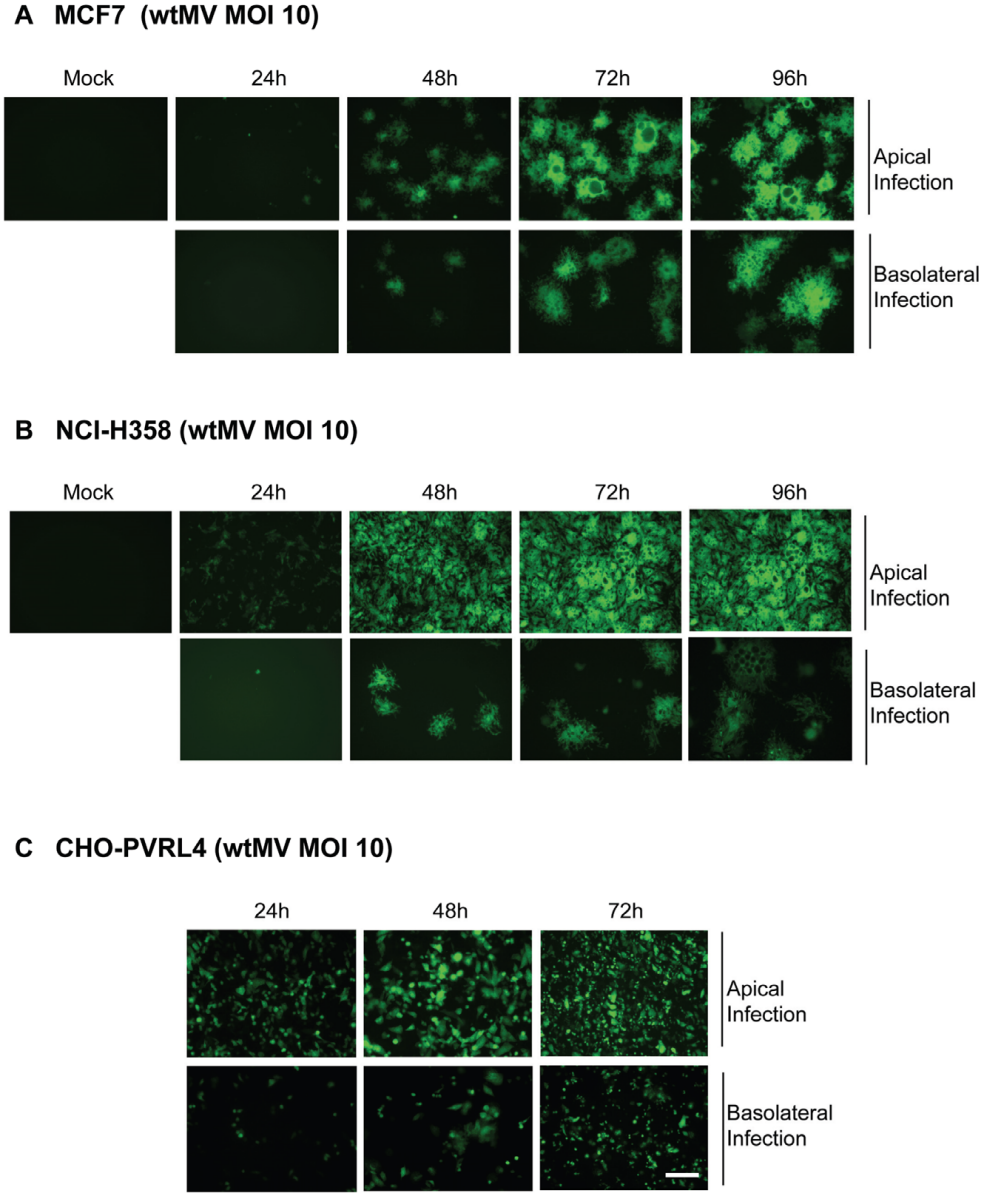
MV infects polarized adenocarinoma cells via either the apical or basolateral surfaces. Wild type IC323 MV infects (A) MCF7 (breast), (B) NCI-H358 (lung) adenocarcinoma and (C) CHO-PVRL4 cell lines via the apical and basolateral surface in Transwell filter assays. Cells were cultivated in Transwell permeable filter supports at a density of 7.0×10^5^ cells per Transwell filter (24 mm diameter) for 4 days (MCF7 & NCI-H358) or 2 days (CHO-PVRL4). Cells were then infected from either the apical or basolateral side with IC323-EGFP wtMV. At various times post infection fluorescent images were captured. Scale bar = 500 µm.

Many of the epithelial cell lines susceptible to wtMV have previously been shown to be polarized. In order to determine whether the putative epithelial receptor was situated on either the apical or basal surface of the cellular monolayer, cells were cultivated on polyester Transwell filter supports (0.4 µm pore size, 24 mm diameter). Cells were ascertained to be polarized by measuring their transepitheilal electrical resistance (TEER). In uninfected cells the TEER maximized at 1200 Ω-cm^2^ at 4 days and remained constant for 10 days from the time of initial culture. Confluent cell monolayers were infected from either the apical or basolateral side with IC323-EGFP wtMV and visualized by fluorescence microscopy. The virus preferentially infected both MCF7 and NCI-H358 cells via the apical route, although basolateral infection was seen at later times post infection (Figure 5A and 5B). To control for the ability of the virus to traverse through the membrane pores, CHO cells stably expressing PVRL4 were inoculated with wtMV from either the apical or basolateral side of the transwell filter (Figure 5C). These non-polarized cells express PVRL4 on both their apical and basolateral surfaces. A lag in MV replication revealed by EGFP expression was observed in the basolateral infections compared to the apical infections. These data suggest that the Transwell membrane may play a role in hindering the ability of MV to infect cells via the basolateral surface. To increase the efficiency of basolateral infections, we prolonged viral adsorption times to 4 hr and decreased the stringency of washing non-adsorbed virus from the cells, but this had no effect. We concluded that wtMV could infect polarized MCF7 and NCI-H358 cells via either the apical or basolateral route, suggesting that PVRL4 is expressed on both cell surfaces.

**Figure 6:**
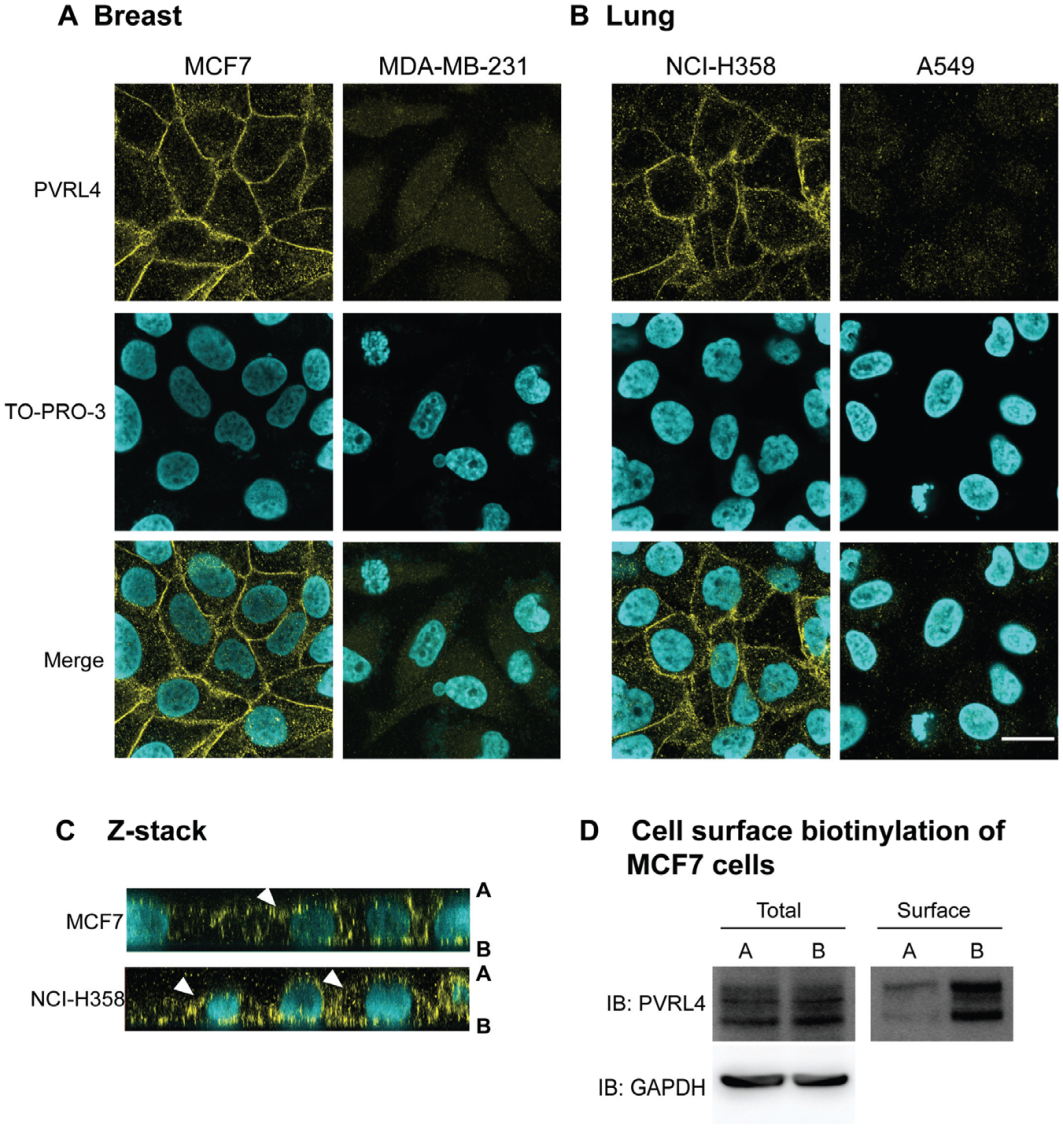
PVRL4 is localized to both the apical and basolateral surfaces in MCF7 and NCI-H358 cancer cells. (A) Breast (MCF7 and MDA-MB-231) and (B) lung (NCI-H358 and A549) cancer cell lines were grown to confluence on glass coverslips and then fixed with paraformaldehye, permeabilzed, and stained with goat-anti human PVRL4 antibodies (yellow). Nuclei were visualized with TO-PRO-3 nuclear stain (cyan). Images were captured on a Zeiss upright confocal microscope and analyzed using Zen 2008 image capture software (Zeiss). Scale bar  = 20 µm. (C) Z-sections of MCF7 and NCI-H358 cells stained with PVRL4 (yellow) and TO-PRO-3 (cyan). PVRL4 is localized to both the apical [A] and basolateral [B] surfaces of these cells. White arrowheads indicate the apical expression of PVRL4. (D) Surface biotinylation of MCF7 cells. MCF7 cells were grown for 96 h on transwell filters (24 mm diameter). The cells were incubated with NHS-biotin from either the apical (lanes A) or basolateral (lanes B) side. After lysis, surface proteins were immunoprecipitated with Neutravidin, and immunocomplexes were subjected to SDS-PAGE and Western blot for PVRL4. Glyceraldehyde 3-phosphate (GAPDH) was used as a loading control.

To investigate the expression pattern of PVRL4 on adenocarcinoma cell lines, susceptible (MCF7 and NCI-H358 cells) and non-susceptible (MDA-MB-231 and A549) cells were stained with PVRL4 antibodies (Figure 6A and 6B). PVRL4 expression was localized to the junctions between cells in susceptible cells only. Upon further examination, PVRL4 appeared to be expressed on both the apical and basolateral side of MCF7 and NCI-H358 cells (Figure 6C & 6D). Surface biotinylation of MCF7 cells also confirmed that PVRL4 was expressed on both the apical and basal surfaces (Figure 6D). Membrane proteins were biotinylated on either the apical or basolateral sides of the cell, precipitated with Neutravidin, resolved by SDS-PAGE, and PVRL4 was detected on immunoblots with specific antibodies. The data confirmed that PVRL4 was expressed on both the apical and basolateral surfaces of adenocarcinoma cell, although the band intensities did not appear to be quantitative. Apical labeling of PVRL4 did not appear to be as efficient as that of the basolateral protein. This may be due to cell surface factors such as mucous formation or glycocalyx, and this observation will require further investigation. However, the biotin labeling studies do confirm qualitatively that PVRL4 is situated on both surfaces of the cell.

PVRL4 is also localized to the cellular junctions of normal and cancerous tissues. The protein is abundantly expressed in most lung adenocarcinomas, some lung squamous carcinomas, an NIC-H358 xenograft from mice, and placenta microvilli. Reactive pneumocytes derived from normal lungs and tonsils exhibited lower levels of expression (Figure S5 in Text S1). Recent reports from The Human Protein Atlas Project (www.proteinatlas.org) have shown that PVRL4 is expressed abundantly in placental trophoblasts, glandular cells of the stomach, and adenocarcinomas of the lung, breast, and ovary. According to this study, moderate amounts of this protein are expressed in the epithelium of tonsils, oral mucosa, esophagus, and the respiratory cells of the nasopharynx. Smaller amounts are expressed in the lung macrophages and neuronal cells of the cerebral cortex.

### siRNA directed against PVRL4 blocks infections by wtMV

**Figure 7:**
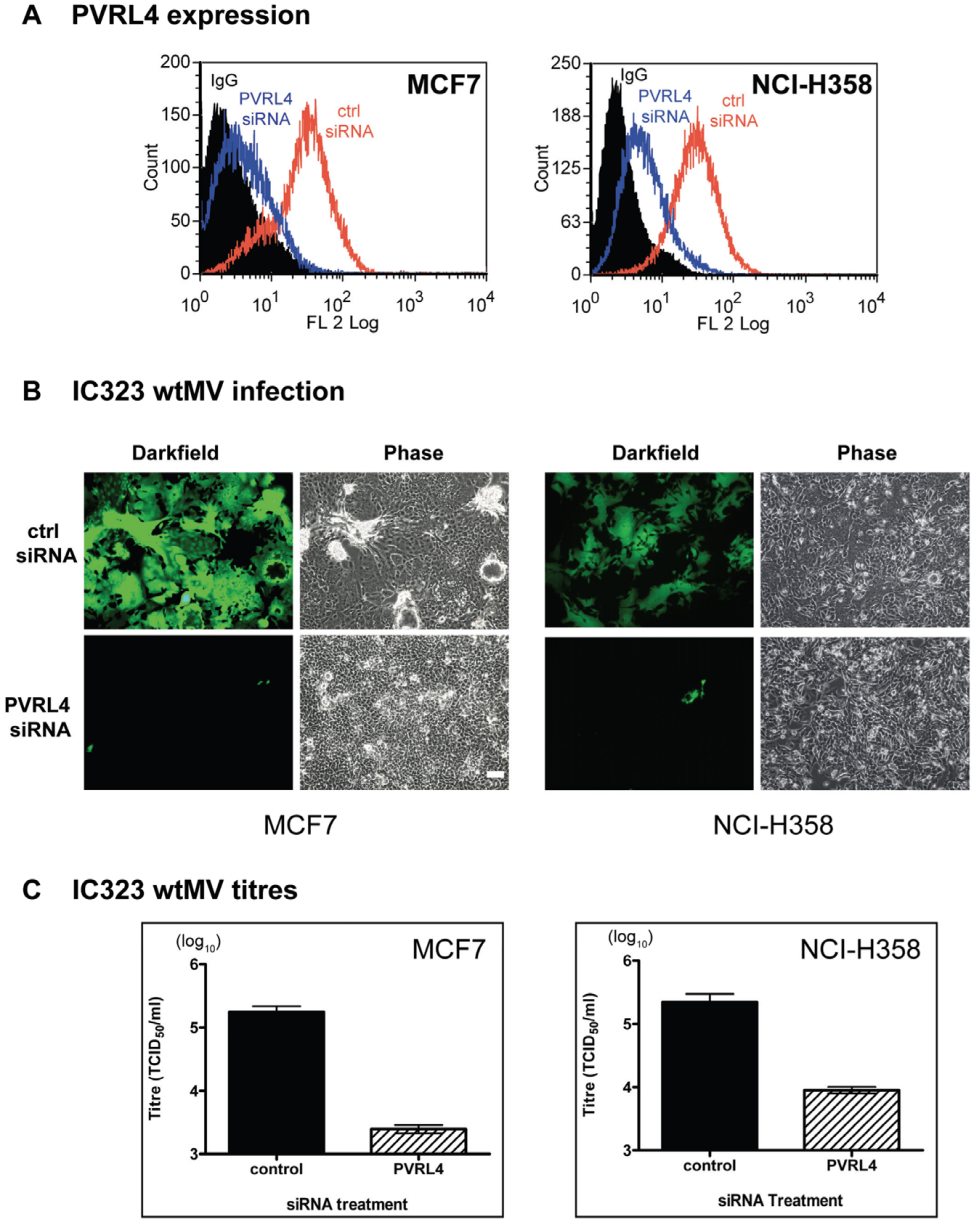
siRNA specific for human PVRL4 inhibits wtMV infections. MCF7 and NCI-H358 cells were transfected with a scrambled oligonucleotide control (ctrl siRNA) or a siRNA pool specific for PVRL4 (PVRL4 siRNA). The transfected cells were incubated with IC323-EGFP wtMV and images were captured 48 hr post infection. (A) PVRL4 surface expression was detected with a phycoerythrin conjugated PVRL4 antibody following gene knockdown with control siRNA (red line) or PVRL4 siRNA (blue line). (B) PVRL4 siRNA-treated MCF7 and NCI-H358 cells showed less GFP expression compared to ctrl siRNA-treated cells. (C) PVRL4 knockdown results in a decrease in wtMV titres in MCF7 and NCI-H358 cells. Forty-eight hours post infection, cells were harvested and TCID_50_ virus titrations were performed on Vero-SLAM cells. Data are the means from three independent experiments, and error bars represent the SEM. Scale bar = 100 µm.

To investigate whether PVRL4 was a *bona fide* receptor for wtMV, siRNA against PVRL4 was used in the susceptible MCF7 and NCI-H358 cell lines. A pool of siRNA specific for PVRL4 or a scrambled siRNA control were transfected into MCF7 or NCI-H358 cells for 72 hrs. FACS analysis demonstrated that PVRL4 surface expression was effectively reduced following siRNA knockdown (Figure 7A). The cells were subsequently infected with IC323-EGFP wtMV and fluorescence was monitored after a further 48 hr incubation, at which point virus was harvested. Scrambled siRNA did not inhibit MV infections (Figures 7B, 7C) while PVRL4 siRNA treatment clearly blocked the fluorescence produced by MV. Virus released from siRNA-treated MCF7 and NCI-H358 cells was subsequently quantified on Vero/SLAM cells. A decrease in approximately 1–2 logs was consistently seen when PVRL4 expression was knocked down prior to MV infection. The siRNA inhibition experiments conclusively demonstrated that unrestricted PVRL4 surface expression was essential for wtMV infection.

### Antibodies specific for human PVRL4 inhibit wtMV infection in MCF7 cells

**Figure 8:**
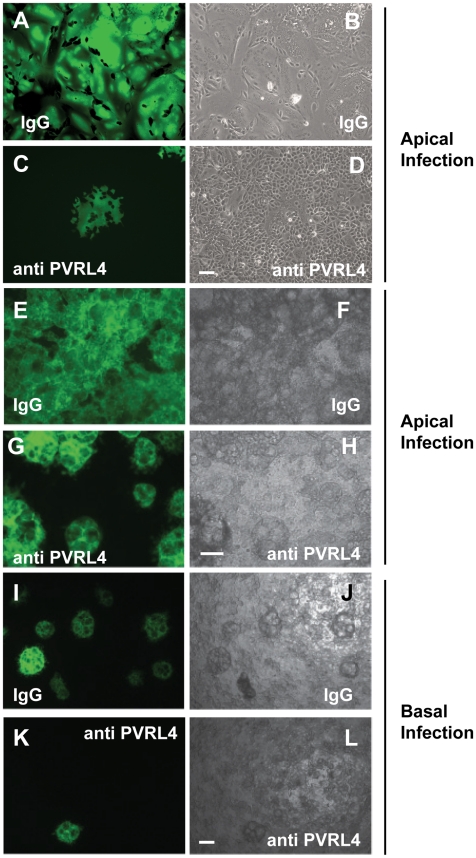
Antibodies specific for human PVRL4 inhibit wtMV infection in MCF7 cells. MCF7 cells grown on glass coverslips were incubated with 10 µg/ml goat IgG (A,B) or goat anti-PVRL4 (C,D) for 30 min prior to, and during 1 hr adsorption with IC323-EGFP MV via the apical surface. Fluorescence and syncytia formation due to viral infection at 48 hrs was inhibited by the PVRL4 antibody treatment. To determine whether PVRL4 antibodies would also inhibit MV infections via the basolateral route, MCF7 cells were grown on Transwell permeable filter supports as described in Figure 5. Cells were incubated with 25 µg/ml goat IgG on the apical (E,F,G,H) or basal (I,J,K,L) surface with antibodies specific for human PVRL4 or non-immune antibodies (IgG) for 30 min. Cells were subsequently inoculated with IC323-EGFP MV (MOI = 10) for 4 hrs, also in the presence of antibody. Infections were allowed to proceed for 72 hrs and cells were viewed by fluorescence and bright field microscopy. The interaction of goat polyclonal antibodies with PVRL4 blocked MV infection of MCF7 cells via either the apical or basal routes. Scale bar  = 100 µm.

MCF7 cells grown on glass coverslips were incubated with 10 µg/ml non-immune goat IgG (Figure 8A and 8B) or goat anti-PVRL4 (Figure 8C and 8D) for 30 min prior to, and during 1 hr adsorption with IC323-EGFP MV via the apical surface. Fluorescence and syncytia formation due to viral infection at 48 hrs was inhibited by the PVRL4 antibody treatment. To determine whether antibodies directed against PVRL4 also blocked infection by the basolateral route, MCF7 cells were grown on Transwell permeable filter supports as described in Figure 5. Cells were incubated on the apical (Figure 8E, 8F, 8G, and 8H) or basal (Figue 8I, 8J, 8K, and 8L) surfaces with antibodies directed against human PVRL4 or non-immune antibodies for 30 min and subsequently inoculated with IC323-EGFP MV (m.o.i. 10) for 4 hrs in the presence of antibody. Infections proceeded for 72 hrs and cells were viewed by fluorescence and bright field microscopy. Interaction of goat polyclonal antibodies with PVRL4 blocked MV infection of MCF7 cells when applied via either the apical or basal routes. This inhibition indicated that MV can infect adenocarcinoma cells in a PVRL4-dependent manner by either the apical or basolateral route. The antibody inhibition provided further corroboration of the preceding RNA interference studies directed against PVRL4.

### PVRL4 acts as an attachment receptor for wtMV

**Figure 9:**
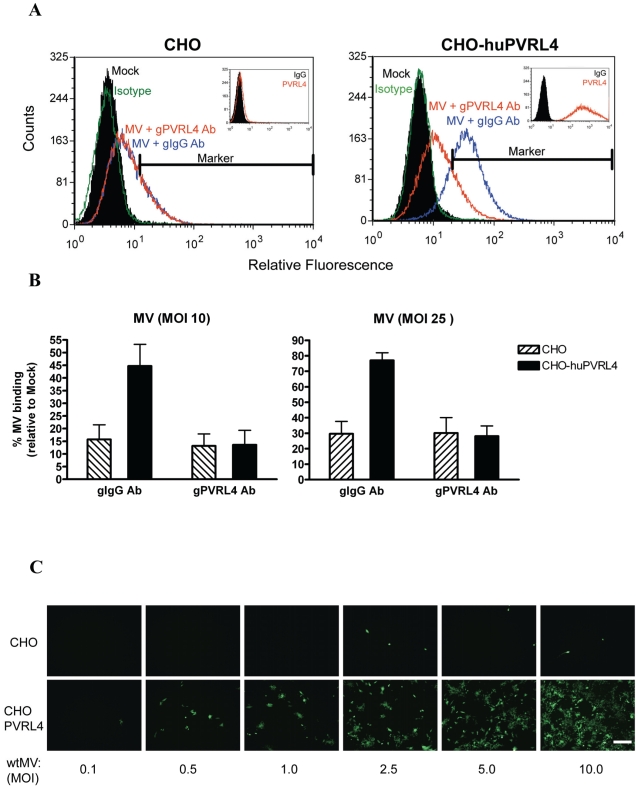
IC323 wtMV binds to cells that stably express human PVRL4. CHO or CHO stably expressing human PVRL4 (CHO-huPVRL4) were incubated with either 10 or 25 PFU/cell of IC323-EGFP wtMV in the presence of isotype (gIgG Ab) or blocking antibodies against PVRL4 (gPVRL4 Ab) for 1.5 h. Cells were incubated with a MV anti-H primary antibody followed by an anti-mouse alexa fluor 488 conjugated secondary antibody to detect MV-bound cells. (A) Binding of IC323 wtMV to cells stably expressing PVRL4 was detected by FACS. CHO and CHO-huPVRL4 cells were inoculated with MV in the presence of blocking antibody against PVRL4 (gPVRL4, red line) or an isotype control (gIgG, blue line), washed, and incubated with anti-MV hemagglutinin antibody or an isotype matched control antibody (green line). Cells incubated in the absence of virus (Mock, filled histogram) were stained with anti-MV hemagglutinin antibody. Bound MV-specific primary antibody was detected with alexa fluor 488-conjugated goat anti-mouse secondary antibody. The relative fluorescence intensity was measured on a Cyan ADP Flow Cytometer. Inset: Receptor expression was detected with a PE-conjugated PVRL4 antibody (red histogram) or isotype control (filled histogram). (B) Quantification of MV binding to CHO cells expressing huPVRL4 in the presence of blocking antibody to PVRL4 (gPVRL4 Ab). The perecentage of MV-bound cells compared to mock cells was determined using FCS express (De Novo software). Data are expressed as the mean from three independent experiments, with error bars showing the SEM. (C) Infection of CHO and CHO-huPVRL4 cells with varying multiplicities of infection using IC323-EGFP wtMV. Images were captured 48 h post infection. Scale bar  = 500 µm.

To assess the ability of MV to bind PVRL4, CHO*pgsA745* cells, which lack heparan and chondroitin sulfate on their surface, were engineered to stably express PVRL4 (CHO-PVRL4). Flow cytometry with a monoclonal antibody specific for human PVRL4 indicated extensive surface expression of this protein on the CHO-PVRL4 cells (Figure 9A, inset). CHO and CHO-PVRL4 cells were incubated with wtMV in the presence of blocking antibodies to PVRL4 (gPVRL4) or an isotype control (gIgG). Virus binding was detected using a monoclonal antibody directed against the H protein and an alexa fluor 488-conjugated goat anti-mouse secondary antibody. Interestingly, background wtMV binding was consistently ∼15–30% in CHO cells irrespective of whether the blocking antibody to PVRL4 was present (Figure 4A, CHO; Figure 9B). In CHO-PVRL4 expressing cells, however, there was a shift in the histogram peak in the gIgG + wtMV treatment, indicating that wtMV had bound to these cells (Figure 9A). When blocking antibodies to PVRL4 were present, the MV binding decreased to background levels seen in the CHO cells (Figure 9B, compare CHO-huPVRL4 gIgG Ab to gPVRL4 Ab) irrespective of the MOI used. These data suggest that PVRL4 is an attachment receptor for wtMV. The CHO-PVRL4 cells were subsequently infected with various multiplicities of infection (MOI) of IC323-EGFP wtMV for 48 h (Figure 9C). An increase in the level of wtMV replication was detected with increasing amounts of MV in the CHO-PVRL4 cells, but only background infections were seen in the CHO cells lacking PVRL4. These results clearly establish PVRL4 as an attachment receptor for MV.

### Mouse PVRL4 functions less efficiently as a receptor for MV than the human homologue

**Figure 10:**
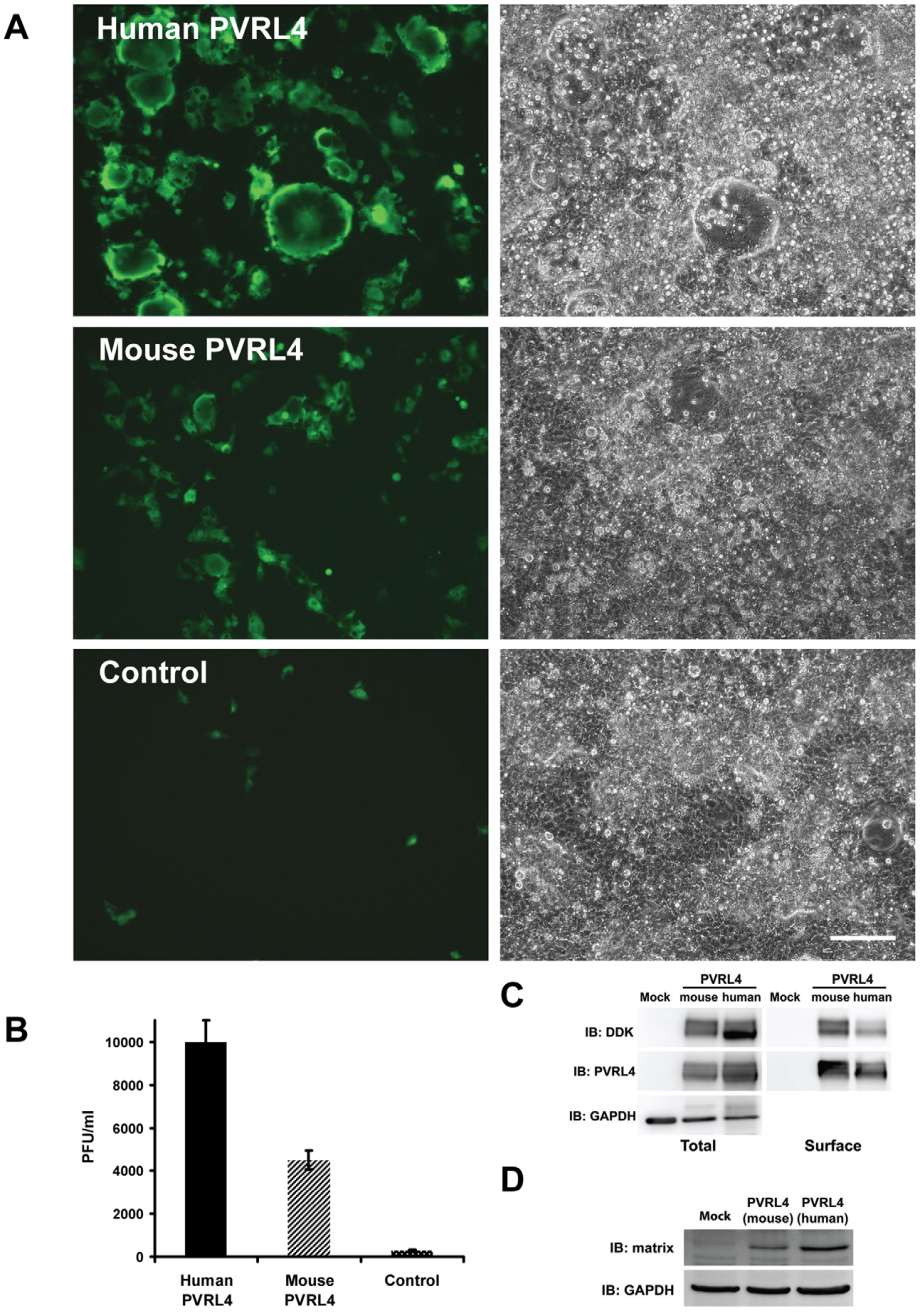
Mouse PVRL4 functions less efficiently as a MV receptor than the human homologue. COS-1 cells were transfected with expression vectors encoding DDK-tagged human and mouse homologues of PVRL4. Control cells were transfected with empty plasmid. After 36 hrs, the transfected cells were infected with IC323-EGFP wtMV and incubated a further 48 hrs. (A) Cells were viewed by fluorescence and phase contrast microscopy. Scale bar  = 200 µm. (B) Virus released from the infected cells was quantified by plaque assay. Data are expressed as the mean from four independent experiments, with error bars showing the SEM. (C) Total and cell surface expression was evaluated by Western immunoblots using antibodies directed against the DDK(Flag) tag or PVRL4. Surface expression was evaluated following biotinylation of plasma membrane proteins. (D) Viral proteins were synthesized in PVRL4 transfect cells following MV infection as shown by Western immunoblot using an antibody specific for the viral matrix (M) protein.

Mouse PVRL4 shares 92% amino acid sequence identity with the human homologue (Figure S6 in Text S1). Expression vectors containing the cDNA sequences for the Myc-DDK tagged versions of human and mouse PVRL4 were transfected into COS-1 cells. These cells were infected with IC323-EGFP wtMV and viewed by fluorescence microscopy at 48 hrs post-infection (Figure 10A). COS-1 cells expressing mouse PVRL4 were less susceptible to infection by IC323-EGFP wtMV and produced smaller and fewer syncytia than cells transfected with the human homologue (Figure 10A). Virus released from the infected cells was compared using quantitative plaque assays. As expected, COS-1 cells transfected with mouse PVRL4 produced less MV than cells transfected with the human PVRL4 homologue (Figure 10B). These results were consistent over the course of 4 separate experiments. Expression levels of mouse PVRL4 were compared to human PVRL4 by immunoblot analysis with antibodies specific for the Myc-DDK tags and were found to be similar. Surface expression of mouse and human forms of PVRL4 were also comparable (Figure 10C). Finally, MV proteins were synthesized in the infected cells as shown by a Western immunoblot using antibodies directed against the matrix (M) protein (Figure 10D).

### Other MV strains can also use PVRL4 (Nectin 4) as a receptor

Other strains of MV were tested for their ability to use PVRL4 as a cellular receptor. The Edmonston-EGFP vaccine strain (Figure S7A and S7B in Text S1), WTF-EGFP wtMV (Figure S7C and S7D in Text S1), and Montefiore 89 wtMV (Figure S7E and S7F in Text S1), were inoculated onto cells transfected with the human PVRL4 expression vector. In the case of Edmonston-EGFP MV, we chose to use owl monkey kidney (OMK) cells, which are known to be deleted for the critical SCR1 domain of CD46, and are normally resistant to infection by vaccine strains of MV [53]. The WTF-EGFP wtMV and Montefiore 89 wtMV cannot use CD46 as a receptor, and were inoculated onto HeLa and 293 HEK cells, respectively, that expressed PVRL4. In both experiments, expression of PVRL4 converted the non-susceptible OMK and COS-1 cells to a MV susceptible phenotype. Cells infected with Montefiore 89 wtMV were fixed with paraformaldehyde and incubated with antibodies specific for MV proteins (H, M). Infections were detected by EGFP fluorescence or anti-measles H, M immune fluorescence microscopy (Figure S7 in Text S1).

### PVRL4 surface expression is down regulated in MCF7 cells following wtMV infection

**Figure 11:**
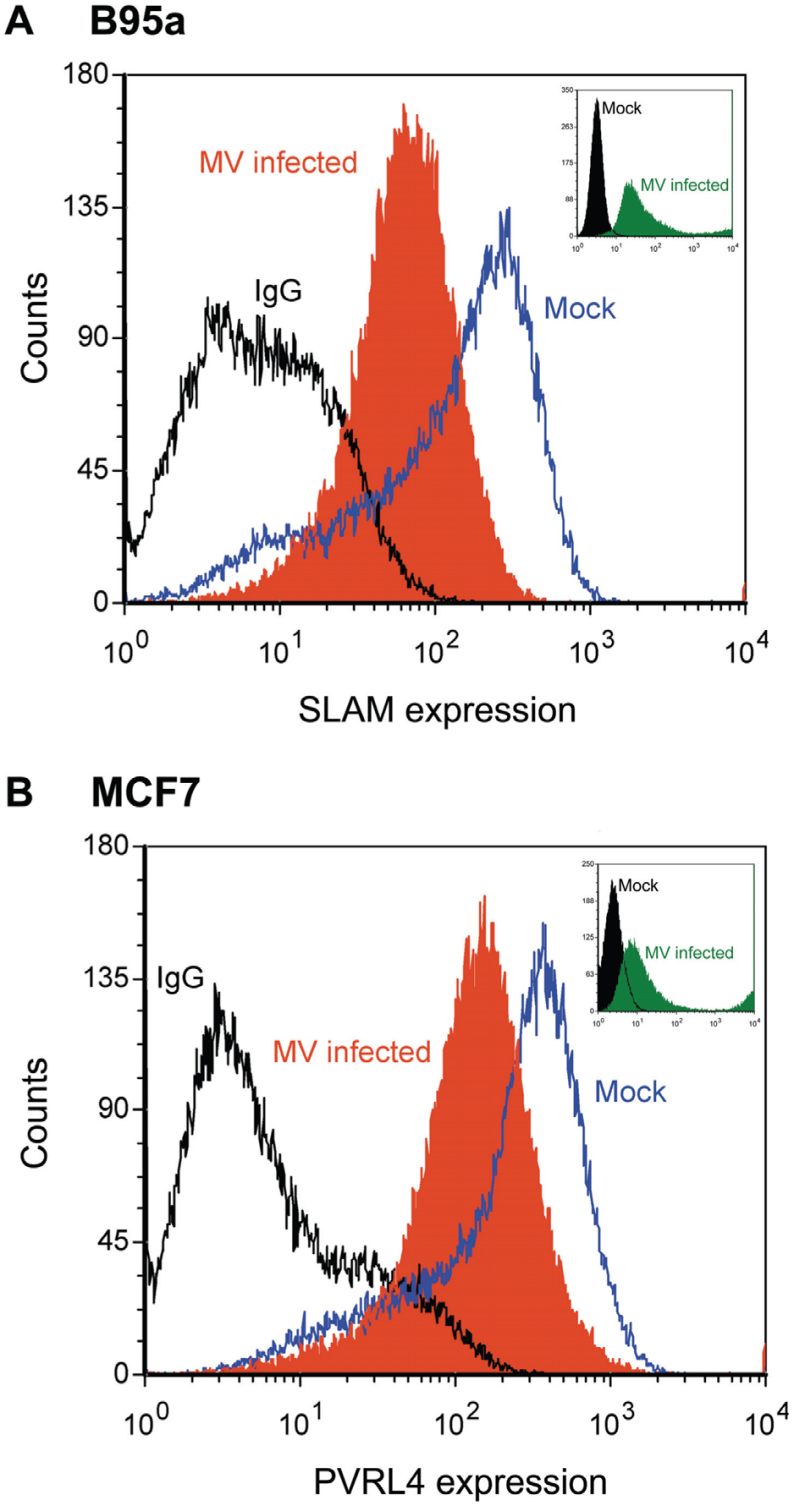
Surface PVRL4 expression is down regulated following wtMV infection. (A) activated marmoset B-cell line B95a or (B) MCF7 cells were infected with IC323-EGFP wtMV. The fusion inhibitory peptide (FIP) was added after the initial virus infection to prevent syncytia formation. At 48 h post-infection SLAM and PVRL4 surface expression was analyzed by FACS. Blue lines, mock-infected cells stained with alexa anti-SLAM antibody (A) or anti-PVRL4 antibody (B); black lines, mock infected cells stained with the anti-mouse IgG2B isotype control antibody; filled orange histogram, cells infected with IC323-EGFP wtMV (MOI = 10) and stained with anti-SLAM (A) or anti-PVRL4 (B) antibodies, respectively. Alexa fluor conjugated 647 secondary antibodies were used to detect SLAM and PVRL4 surface expression. Insets, level of eGFP positive cells following a 48 h infection with IC323-EGFP wtMV. The filled green histogram represents wtMV-infected cells; black lines represent mock-infected cells.

An important aspect of MV infection is the down regulation of CD46 and SLAM from the cell surface following MV-H expression [56]–[60] To determine whether PVRL4 expression was down regulated in a similar manner, FACS analysis of PVRL4 surface expression was performed at 48 h post infection. Alexa fluor conjugated 647 secondary antibodies were used to detect SLAM and PVRL4 surface expression (Figure 11). SLAM surface expression on B95a cells was down regulated following infection with IC323-EGFP wtMV (Figure 11A) in the presence of the fusion inhibitory peptide, as expected. Similarly, IC323-EGFP wtMV infection caused a decrease in the level of PVRL4 surface expression on MCF7 cells (Figure 11B). The level of MV replication was assayed by the presence of GFP positive cells (Figure 11, inset). At 48 h post infection more GFP positive cells were seen in the MV-infected B95a cells compared to the MV-infected MCF7 cells. Taken together, these data suggest that, like SLAM (CD150), PVRL4 is also down regulated following wtMV infection.

## Discussion

PVRL4 (Nectin 4) was demonstrated to be an epithelial receptor for MV. This protein is a member of the poliovirus receptor-like proteins (PVRLs) that are adhesion receptors of the immunoglobulin superfamily [61]. It is a 510-amino acid transmembrane protein with a predicted molecular mass of 55.5 kDa which migrates with a mass of 66 kDa on SDS polyacrylamide gels due to N-glycosylation. PVRL4 is an embryonic protein which has recently been shown to be a tumor cell marker for lung, breast, and ovarian adenocarcinomas [62]–[64]. Like other nectins, PVRL4 is normally localized to the adherins junctions together with the cadherins. PVRL4 interacts with itself and the V domain of PVRL1, but not with other members of the same molecular family. Its cytoplasmic tail associates with the intracellular actin-binding protein, afadin [65]. In humans it is expressed abundantly in the placenta and weakly in the trachea. However, in the adult mouse, PVRL4 transcripts were also found in the brain, lung, and testis [61]. More recently, The Human Protein Atlas Project (www.proteinatlas.org) has reported that PVRL4 is expressed abundantly in placental trophoblasts, glandular cells of the stomach, and adenocarcinomas of the lung, breast, and ovary. Moderate amounts of this protein appear to be expressed in the epithelial cells of tonsils, oral mucosa, esophagus, and the columnar epithelial cells of the nasopharynx and trachea. Smaller amounts are expressed in the lung macrophages and neuronal cells of the cerebral cortex.

The nectins can also function as entry receptors for several other viruses. PVR (CD155) is the prototype member of the family and was originally shown to be the receptor for poliovirus [66]. PVRL1 (Nectin 1) serves as an entry receptor for herpes simplex virus (HSV). It is the major HSV receptor and mediates entry of all HSV-1 and HSV-2 strains as well as animal alphaherpesviruses [48]. PVRL2 (Nectin 2) can also function as an entry factor for some herpesviruses [67]. Alternatively, HSV can use another receptor of the TNF family called herpes virus entry molecule (HVEM). The fact that HSV-1 and HSV-2 use multiple receptors for cell entry appears to enable the virus to enter different cell types [68]. This also appears to be the case with MV. The expression of Nectin 1 on the cell surface of tumors is also a predictor of oncolytic sensitivity to HSV in potential cancer therapy [69]. Tight junction proteins can also serve as receptors for some viruses. For example, occludin is a major component of tight junctions that is used by hepatitis C virus [50] and group B coxsackievirus as an entry factor [49]. However, occludin shRNA experiments in MCF7 cells had no effect upon MV infectivity (data not shown).

The actual role of epithelial cells in MV infections has been controversial in light of recent experiments with macaques and SLAM transgenic mice. Alveolar macrophages, dendritic cells, and activated lymphocytes have recently been reported to be primary targets for IC323-EGFP wtMV and rMV^KS-^EGFP strains, and there was only limited infection of epithelial cells in the studies with macaques over 5 to 9 days infection [32]–[35]. Although lymphocytes and dendritic cells may be the major target and reservoir, virus has also been reported in the squamous stratified epithelium of the tongue and buccal mucosa and in the ciliated epithelium of the trachea at the peak of infection [32]. It has been proposed that immune cells transmit MV to airway epithelial cells via a receptor on their basolateral surface and that these infected cells release virus from their apical cell surface to further infection and spread [33], [42]. It is possible that infected lymphocytes, expressing H and F proteins on their surface, attach to PVRL4 expressing epithelial cells to facilitate cell to cell spread of MV and syncytia formation late in infection. Again infected epithelial cells appeared at later times of infection than infected lymphocytes and dendritic cells [70]. When a mutant MV that was blind to the epithelial receptor was used to inoculate macaques, clinical symptoms of measles were observed, but no virus was shed into the airways of these animals [33]. This observation has led the researchers to conclude that the infection of epithelial cells occurred later in the disease, and was important for aerosol transmission of the virus.

We were initially surprised to observe that wtMV could infect adenocarcinoma cell lines from the apical surface in our Transwell filter assays. However, this finding may not be unreasonable since we were working with cancer cells where PVRL4 is highly upregulated, and it is expressed on both apical and basolateral cell surfaces. We have carefully considered the possibility that MV infection of adenocarcinoma cell lines using PVRL4 may not be totally relevant to understanding viral pathogenesis in the airways of the normal host. It has previously been reported that MV preferentially infects differentiated primary epithelial cells via the basolateral route, which is consistent with the location of PVRL4 at the adherens junctions of normal cells [33], [34], [40]. Foci of infected cells derived from basolateral infections of primary epithelial cells did not appear to fuse and produce syncytia. This also warrants explanation. In addition, we and others were able to convert primary human SAEC grown in serum free to a MV susceptible phenotype by culturing them in 2% fetal calf serum. Flow cytometry indicated that little PVRL4 was produced on SAEC grown in serum free media, but the nectin was induced following transfer to serum containing media. PVRL4 may be expressed at higher levels on the apical surface during phases of rapid growth. In support of this, PVRL4 is highly expressed during embryogenesis [61]. Upon revisiting the literature, we discovered that laboratories looking at basolateral infections of polarized primary human epithelial cell monolayers were waiting from 3–7 days post infection in order to see infected cell foci, and that apical infection of susceptible cancer cell lines was more efficient. This was consistent with our observations in Figures 5 and 8. In our hands, both MCF7 and NCI-H358 cells produced syncytia when infected via the basolateral route, although apical infection was much more efficient. Similar results were reported by Tahara *et al*. who showed that polarized CaCo2 and HT-29 cells were preferentially infected via the apical surface [41]. They also commented in their results section that the virus infected the basolateral side of the polarized monolayer much less efficiently than the apical side. Other investigators also obtained similar results using a laboratory strain of MV to infect CaCo2 cells [40]. However, when they infected primary human airway epithelia via the basolateral surface, they saw more virus replication compared to infection of the apical side. Mutations in the putative epithelial receptor binding region of MV H protein, to produce an epithelial receptor blind virus, blocked basolateral infection of primary cells as well as apical infection of lung adenocarcinoma cell lines, indicating that the same receptor is probably used in each case [33], [41].

Other laboratories have shown that PVRL4 (Nectin 4) is up-regulated on breast, lung, and ovarian cancer tumors and cell lines [62], [63], [71]. We extended this observation to colon carcinoma cell lines including DLD-1, HT29, and LoVo cells. There are anecdotal reports in the literature where natural MV infections were shown to reverse cases of Burkitt's lymphoma and Hodgkin's disease [72]–[75]. Given that these tumors express SLAM/CD150, one can presume that wtMV infected the tumors and triggered immune attack against them. Vaccine strains of MV have previously been engineered to recognize cancer cells by artificially manipulating the H receptor binding protein [12]. Clinical trials are currently in progress at the Mayo Clinic (Rochester, MN) for ovarian cancer, pancreatic cancer, glioblastoma, medulloblastoma, and multiple myeloma [12]. It may be possible that a natural tropism of MV for lung, breast, colon, bladder, and ovarian adenocarcinomas can also be exploited for future oncolytic therapies. The use of SLAM/CD46 blind MV that retained the ability to bind PVRL4 could constitute a potential therapeutic vaccine against adenocarcinoma. Safety issues, background infection, and the effect of pre-existing antibodies from MMR vaccination will obviously have to be addressed before human clinical trials could even be considered.

This study identifies PVRL4 (Nectin 4) as an epithelial receptor for MV. PVRL4 is expressed at low to moderate levels in normal tissues but is highly up-regulated on the surfaces of adenocarcinoma cells. The interaction is highly specific, since MV does not recognize other members of the PVRL family and prefers the human receptor over homologues in the mouse. Further experiments with differentiated primary epithelial cells in culture and the use of human epithelial explants will be required to validate the role of PVRL4 in infections of normal epithelial cells and establish its importance in measles pathogenesis. These studies are currently underway in our laboratory.

## Materials and Methods

### Antibodies

M75 and B97 monoclonal antibodies, which neutralize CD46 binding to MV, were obtained from Seikugaku (Tokyo, Japan) and Dr. J. Schneider-Schaullies (Wurzburg, Germany), respectively. IPO-3 and A12 monoclonal antibodies, which inhibit CD150 binding to MV, were purchased from AbCam (Cambridge, MA). PE-conjugated mouse anti-human CD150/SLAM (clone A12) and PE-conjugated mouse IgG1 kappa isotype control (clone MOPC-21) were from BD Biosciences. Unconjugated mouse anti-human nectin-4 (MAB2659), PE-conjugated mouse anti-human nectin-4 monoclonal (FAB2659P), PE-conjugated mouse IgG2B isotype control (IC0041P), goat polyclonal anti-human PVRL4 (AF2659), and control goat (AB-108-C) antibodies came from R&D Systems (Minneapolis, MN). Monoclonal mouse anti-V5 (Sigma, clone V5-10) was used to detect V5 tagged proteins synthesized from the pcDNA3.2 DEST/V5 expression vector. The anti-Flag antibody (Sigma) was used to detect DYKDDDDK tagged proteins expressed from the pCMV6 entry vector.

### Cell culture and virus infections

Human primary small airway epithelial cells (SAEC) were obtained from Lonza Walkersville Inc., (Walkersville, MD). Marmoset SAEC were prepared by the custom service division of Lonza Walkersville Inc. Vero, B95a, OMK, HeLa, LoVo, Huh7, HepG2, Hep3B, and CHO*pgsA745* cells, were purchased from the American Type Culture Collection (Manassas, VA). NCI-H125, NCI-H157, NCI-H460, SBC-3, NCI-H661, NCI-H520, RVH6847, NCI-226, MGH-7, MGH-24, and NCI-H358 cells came from Dr. Ming-Sound Tsao (Ontario Cancer Institute, Toronto, Canada). MDA-MB-468, MDA-MB-231, MCF7, T47D, HT-29, T84, HCT116, HS766T, DLD-1, and MDCK cells were acquired from Drs. David Hoskin and Craig McCormick (Dalhousie University, Halifax, Canada). The Edmonston vaccine/laboratory strain of MV was originally obtained from Dr. Erling Norrby (Karolinska Institute, Stockholm, Sweden). The recombinant Ichinoise-B 323 (IC323) wild type isolate expressing EGFP reporter gene (IC323-EGFP wtMV) and a recombinant Edmonston MV containing a WTF H protein (in place of the H protein of the vaccine strain), Edmonston-EGFP MV, SLAM blind-EGFP and CD46 blind-EGFP recombinant viruses were obtained from Dr. Roberto Cattaneo [33], [52]. The Montefiore 89 strain of MV (wild type) was obtained from Ilya Spigland and Amy Fox (Montefiore Medical Center, Bronx, NY).

### CD46 diagnostic RT-PCR and agarose gel electrophoresis

Total RNA was extracted from HeLa, marmoset kidney NZP60, and marmoset SAEC using TRIzol (Invitrogen). First strand cDNA was prepared with a SuperScript III kit (Invitrogen). PCR was performed with conserved diagnostic CD46 primers spanning the SCR1 coding region of cDNA from the different cell types [5′oligo: gccgccgcgagtgtccctttccttc; 3′oligo: cactttggaactgggggatcccaag]. PCR amplification was done using *PFUultra* II fusion HS polymerase (Stratagene). A 50 µl reaction volume was initially heated for 2 min at 95°, processed through 30 cycles of sequential temperatures of 95° (30 sec), 58° (30 sec), 72° (30 sec) and finally incubated for 10 min at 72°, using an Applied Biosystems Geneamp 9600 PCR machine. Samples were stored at 4°, prior to electrophoresis at 120 V on 0.9% agarose gels containing ethidium bromide. The PCR product derived from full length human CD46 cDNA was 834 bp and that from marmoset CD46 cDNA was 645 bp, as predicted from the sequences in the NCBI genebank (NM_002389.4 and U87917).

### Microarray analysis

Primary SAEC (Lonza) were cultured in a 6-well culture plate in DMEM with and without 2% FCS for 22 hrs. Cell lines were grown in 75 cm^2^ T-flasks containing DMEM and 10% FCS. Extraction of total RNA was performed using a Qiagen RNeasy Kit (Qiagen). Analysis for mRNA transcripts was performed using the Affymetrix Human Gene ST 1.0 Array at The Centre for Applied Genomics located at The Hospital for Sick Children in Toronto, Canada. cDNA's from SAEC, susceptible (MCF7, MDA-MB-468, T-47D, NCI-H125, NCI-H358, and MGH-24) and non-susceptible (A549, MDA-MB-231) cell lines were biotin labeled, hybridized to the microarray chip, washed, and stained with streptavidin-PE. Normalized probe set data was analyzed with the Affymetrix Expression Console 1.1 software. Microarray data was deposited in the NCBI GEO database (accession #GSE26636). Further details are provided in Supporting Information.

### Plasmid transfection of candidate epithelial receptors

A human plasma membrane open reading frame gene collection (HS5016) was obtained from Open Biosystems (Huntsville, AL). The genes contained within pDONR223 entry vectors were introduced into the Gateway pcDNA3.2/V5-Dest mammalian expression plasmid through recombination using the LR Clonase II system (Invitrogen). These genes contained a V5 tag. Genes which were not contained in the Open Biosystems Membrane Protein collection were purchased from Origene Systems (Rockville, MD) and contained a DDK (Flag) tag. Expression plasmids were introduced into non-susceptible cells using Lipofectamine 2000 (Invitrogen) according to the manufacturer. Empty vector (pcDNA3.2-V5/Dest or pCMV6 DEST) as well as EGFP and SLAM expressing plasmids were included as controls. At 36–48 hrs post transfection, cells were inoculated with IC323-EGFP wtMV in Opti-MEM media (Invitrogen) at an m.o.i. of 10 for 2 hrs at 37°C. The inoculums were replaced with Dulbecco's minimum essential media containing 2% fetal calf serum. After 48 hrs, infected cells were visualized by phase contrast and fluorescence microscopy.

To assess protein expression of the candidate receptors, cell monolayers were lysed in radioimmunoprecipitation (RIPA) buffer (50 mm Tris-HCl, pH 7.4, 1% NP-40, 0.25% sodium deoxycholate, 150 mM sodium chloride, 1 mM ethylenediaminetetraacetic acid, 1 mM sodium fluoride, 1 mM sodium orthovanadate, 1 mM phenylmethylsulfonyl fluoride, 2 mM dithiothreitol, 1x protease inhibitor cocktail [Roche]) for 15 min on ice. The lysate was centrifuged at 13,000 x *g* for 15 min at 4°C, and protein quantification was performed with the Bradford assay kit (Thermo Scientific). SDS-PAGE and Western immunoblotting was carried out using antibodies against DDK and V5 to detect expression of the candidate membrane receptors.

### Flow cytometry

Cells were grown to confluence in 10 cm^2^ dishes, washed twice in cold PBS, and harvested in non-enzymatic cell dissociation buffer (Sigma). 250,000 cells were blocked with 2.5 µg of normal human IgG (R&D Systems) for 10 minutes on ice followed by the addition of 10 µl of either PE-conjugated PVRL4 (R&D Systems FAB2659P) or PE-conjugated mouse IgG2B isotype control (R&D Systems IC0041P) antibodies for 45 min on ice. Cells were washed twice in PBS containing 1% BSA, 5 mM EDTA, and 0.1% sodium azide and then fixed in 1% paraformaldehyde. Samples were run on a Cyan ADP Flow Cytometer (Beckman Coulter) and data were processed using FCS Express (De Novo Software). Unconjugated SLAM and mouse anti-human PVRL4 antibodies were used in the receptor down regulation experiments. Secondary antibodies conjugated to Alexa Fluor 647 were used to detect surface expression of SLAM and PVRL4 using the FL8 channel on the Cyan ADP Flow Cytometer.

### Infection of the basolateral and apical epithelial cell surface with MV

MCF7, NCI-H358, and CHO-PVRL4 cells were seeded onto Transwell permeable filter supports (Corning Inc., 0.4 µm pore size, 24 mm diameter) at a density of 7.0×10^5^ cells per well for 4 days (MCF7 & NCI-H358) or 2 days (CHO-PVRL4). Polarization of MCF7 cells was verified by measuring transepithelial electrical resistance (TEER) with a Millipore-ERS Voltohmmeter equipped with STX electrodes (Millipore, Billerica MA). An impedence of greater than 500 Ω-cm^2^ indicated that a cell line was polarized. To infect the apical surface, 10 PFU/cell of IC323-EGFP wtMV was added to the upper chamber of the transwell filter and allowed to adsorb for 2 h. To infect the basolateral surface, filter inserts were inverted and the virus was adsorbed for 2 h. The virus innoculum was subsequently removed from the apical or basolateral surface and the membranes were treated with citrate buffer to inactivate any non-internalized virus. The transwell filters were then returned to their normal orientation. Infected cells were viewed by fluorescence and phase contrast microscopy using a Leica DMI4000B inverted microscope (Leica Microsystems).

### Confocal microscopy

Cells grown on poly-D-lysine (Sigma) coated coverslips were fixed in 4% paraformaldehyde (10 min) and permeabilzed with 0.1% Triton X-100 in PBS (10 min). PVRL4 was detected by incubating the cells with goat anti-human PVRL4 (R&D Systems AF2659) at 7.5 µg/ml in PBS containing 5% FCS for 45 min at room temperature. Cells were subsequently stained with fluorophore-conjugated secondary antibodies for 30 min at room temperature. Nuclear DNA was stained (20 min) with TO-PRO-3 stain (Invitrogen). Cells were mounted with fluorescent mounting medium and images were acquired with ZEN 2008 imaging software on a Zeiss LSM 510 upright laser scanning confocal microscope. Images were captured with a 100x Plan APOCHRMOAT (1.4NA) objective lens and processed using ZEN 2009 light and Adobe Photoshop CS3 using only linear adjustments.

### Surface biotinylation

Levels of PVRL4 on the cell surface of MCF7 cells were determined by surface biotinylation. Cells were seeded onto transwell filters (0.4 µm pore size, 24 mm diameter) at a density of 5.0×10^5^ cells per filter. Five days post seeding, cells were washed either the apical or basolateral side of the membrane was incubated for 1 hour with PBS containing 2 mM S-NHS-biotin (Thermo Scientific) at 4°C, while 0.1 M glycine was added to the opposite side of the membrane. After washing with 0.1 M glycine, filter membranes were cut and cells were lysed in RIPA buffer Cell lysates were clarified by centrifugation at 21 000 x *g* and biotinyalted surface proteins were immunoprecipitated with agarose-conjugated NeutrAvidin (Thermo Scientific). Following SDS-PAGE and immunoblotting onto polyvinylidene fluoride (PVDF) (Millipore), proteins were detected with goat anti-human PVRL4 antibodies (R&D Systems). Secondary antibodies were conjugated to horseradish peroxidase and visualized by chemiluminescence. Thirty micrograms of total whole cell lysate was run and blotted with anti-human PVRL4 antibodies and anti-GAPDH antibodies to control for protein loading.

### siRNA inhibition

siRNA duplexes against human PVRL4 were purchased from Dharmacon using a predesigned ON-TARGET plus SMARTpool siRNA (L-004301-00-0005). Non-targeting siRNA was used as a negative control (D-001810-10-05). MCF7 and NCI-H358 cells were plated at 30–40% confluence in 35-mm dishes a day before siRNA transfection. One hundred picomoles of siRNA were mixed with 5 µl of Lipofectamine 2000 (Invitrogen) in 500 µl Opti-MEM (Invitrogen) and added to cells in 500 µl Opti-MEM. Cells were transfected at 0 hrs and 10 hrs and incubated an additional 16 hrs. At 26 hrs, Opti-MEM was replaced with DMEM containing 5% FCS and cells were allowed to grow for an additional 48 h, and at 74 hrs into the experiment, cells were again transfected with siRNA and incubated another 18 hrs. At 92 hrs into the experiment, cells were inoculated with IC323-EGFP wtMV at an m.o.i of 5 for 2 hrs. Following adsorption of virus, cells were treated with citrate buffer to remove non-internalized virus, washed 3 times with PBS, incubated with DMEM containing 5% FCS at 37°C for an additional 36 hrs, and viewed by fluorescence and phase contrast microscopy and then harvested to determine MV titres.

### Virus titration

MV-infected cell monolayers were harvested in media and subjected to one freeze-thaw cycle to release virus particles. TCID_50_ titres were determined by 50% end-point titration on Vero/hSLAM cells according to the Spearman-Kärber method. Plaque assays using SeaPlaque agarose overlays were performed as previously described [76].

### MV binding assay

CHO*pgsA745* cells that stably expressed PVRL4 were generated from the pCMV6 AC-PVRL4 expression vector which contained a neomycin^R^ selection marker. Cells were pre-treated with 15 µg/ml of either blocking PVRL4 antibody (R&D Systems AF2659) or an isotype control antibody (R&D Systems AB-108-C) for 30 minutes at 4°C. To assess the binding capacity of MV to PVRL4, CHO-PVRL4 cells were incubated with either 10 or 25 PFU/cell of MV-IC323 for 90 minutes on ice in the presence of isotype (gIgG) or blocking PVRL4 (gPVRL4) antibodies. Cells were washed three times with PBS containing 1% bovine serum albumin, 5 mM EDTA, and 0.1% sodium azide, and incubated with an anti-MV hemagglutinin antibody (Millipore MAB8905) on ice for 60 minutes. The cells were washed prior to incubation with an alexa fluor 488-conjugated goat anti-mouse antibody for 45 minutes on ice. Cells were again washed to remove any unbound antibodies, fixed in 1% paraformaldehyde, and run on a Cyan ADP Flow Cytometer (Beckman Coulter). Data were processed using FCS Express (De Novo Software). To determine the percentage of cells that had MV bound to their surface, a marker was drawn on the histogram so that the percentage of MV-bound cells in the mock sample was 1%. All samples were compared to mock. Data were graphed using GraphPad 4.0 software.

### PVRL4 down regulation following IC323-EGFP wtMV infection

B95a and MCF7 cells were seeded in 6-well plates at a density of 1.5×10^6^ and 7.0×10^5^ cells per well, respectively. Cells were allowed to grow for 24 h and then infected with IC323-EGFP wtMV at 10 PFU/cell for 1.5 h. The virus innoculum was replaced with DMEM containing 5% FCS and 100 µM of the fusion inhibitory peptide, ZDfFG (Sigma C9405) to prevent syncytia formation. Forty-eight hours post infection, cells were harvested in non-enzymatic cell dissociation buffer (Sigma) and stained for SLAM expression using SLAM antibody (BD Biosciences) or PVRL4 expression as described above. Samples were run on a Cyan ADP Flow Cytometer (Beckman Coulter) and data processed using FCS Express (De Novo Software).

### Accession number

Microarray data was deposited in the NCBI GEO database (accession #GSE26636).

### Ethics statement

The experiments in this article were performed at Biological Safety Level 2 in accordance with the regulations set forth by Public Health Agency of Canada and the Canada Food and Drug Inspection Agency. This work did not involve experimentation with animals or human beings.

## Supporting Information

Text S1 Supporting figures, legends, and methods. Figures S1-S7 are presented along with corresponding legends. Supplementary methods that further describe the microarray analysis and immune histology of human tissue sections using PVRL4 antibodies are included.
(PDF)

Table S1 Excel file showing normalized probe intensity values and % up-regulation of membrane protein expression in breast, lung, and primary SAEC. Related to Table 2 showing data used to determine common gene products that were >20% up-regulated in susceptible breast adenocarcinoma, lung adenocarcinoma, and serum activated SAEC.
(XLSX)
