# Supplementary material for: Tumor Cell Marker PVRL4 (Nectin 4) Is an Epithelial Cell Receptor for Measles Virus
Source: PLoS Pathog. 2011 Aug 25;7(8):e1002240. doi: 10.1371/journal.ppat.1002240 (PMC3161989; doi:10.1371/journal.ppat.1002240)
Supplement: Text S1 — Supporting figures, legends, and methods. Figures S1-S7 are presented along with corresponding legends. Supplementary methods that further describe the microarray analysis and immune histology of human tissue sections using PVRL4 antibodies are included. (PDF) [file ppat.1002240.s001.pdf]

Supporting Information

Supporting Figures and Legends

Figure S1. Human SAEC grown in 10% fetal calf serum and many adenocarcinoma cell lines are susceptible to infection by IC323-EGFP wtMV. MGH24 (lung), NCI-H358 (lung), RVH6847 (lung), MCF7 (breast), MDA-MB-468 (breast), T47D (breast), Huh7 (liver) adenocarcinoma cell lines and SAEC (with serum) were visibly infected with the IC323-EGFP wtMV virus after 48 hrs incubation. SAEC (serum free), A549 (lung adenocarinoma), SBC-3 (small cell lung carcinoma), MDA-MB-231 (breast adencarcinoma), and HeLa (cervical carcinoma) were non-permissive for wtMV infections. Scale bar=100μm. See also Table 1.

Figure S2. PVRL4 (Nectin 4) and CD150 (SLAM) expression renders cells susceptible to IC323-EGFP wtMV. Non-permissive OMK, HeLa, A549, and MDA-MB-231 cells were transfected with expression plasmids expressing either CD150/SLAM or PVRL4 (Nectin 4) and incubated for 36 hrs. The transfected cells were infected with IC323-EGFP wtMV (m.o.i. 10) and incubated a further 48 hrs. Scale bar=100μm. See also Figure 3.

Figure S3. Expression of (A) DDK- and (B) V5-tagged candidate receptors in COS-1 cells. COS-1 cells were transfected with expression plasmids containing the coding sequences for candidate membrane protein receptors. After 36 hrs the cells were lysed and 10 μg of whole cell lysate was separated by SDS-PAGE followed by Western immunoblot. Horseradish peroxidase-conjugated Flag antibodies (IB: DDK) or V5 antibodies (IB: V5) were incubated with the membranes and developed with enhanced chemiluminescence. (A) Duplicate expression clones of CDH1, F11R, GPC4, TMEM125, and a single clone of SUSD4 were transfected into COS-1 cells and analyzed with DDK(Flag) antibodies. These clones were purchased from Origene Systems. (B) Expression clones for CLDN1, CLDN4, CLDN7, RAB25, STX6, FAM84, JUP,

TACSTD2, and PVRL2 were prepared from the Open Biosystems Plasma Membrane Donor Library using Gateway Cloning technology and LR Clonase II (see Materials and Methods). The resulting clones contained a V5 tag sequence fused to the coding sequence of a particular gene to produce a recombinant protein with a V5 tag at its carboxyl terminus. See Figure 2 and Table 2.

Figure S4. Comparison of protein sequences for human PVRL1, PVRL2, PVRL3, and PVRL4. Sequences were aligned using the Clustal method from the DNAStar Lasergene analysis software. Shaded residues represent amino acids that are identical to the consensus sequence shared by the 4 proteins. PVRL4 exhibits 38% identity with the consensus. Sequences were obtained from the NCBI GeneBank. [PVRL1 NM_002855.4; PVRL2 NM_002856.2; PVRL3 NM_015480.1; PVRL4 NM_030916.2]. See also Figure 3.

Figure S5. Immune histological analysis of PVRL4 in human tissues. Formalin fixed paraffin embedded tissue slices from placenta, NCI-H358 xenografts grown in mice, lung adenocarcinoma, lung squamous carcinoma, reactive pneumocytes from the lung, and tonsil tissue were incubated with goat anti-PVRL4 antibody (1:1000) directed against a specific peptide sequence. Antibody binding was detected by incubating the tissue sections with biotin-anti-goat IgG, horse-radish peroxidase (HRP)-streptavidin, and diaminobenzidine (DAB) substrate. Labeled PVRL4 protein located in adherens junctions stained brown. Representative lighter staining structures in the pneumocytes and tonsils are indicated with arrows. Scale bar = 100μm. See also Figure 6.

Figure S6. Comparison of protein sequences for human, orangutan (pongo), canine, rat, and mouse PVRL4 (Nectin 4). Sequences were aligned using the Clustal method from the DNAStar Lasergene analysis software. Shaded residues represent differences from the human sequence. Human and orangutan sequences were almost 100% identical. Mouse and rat

sequences were 92% identical to that of humans. The canine PVRL4 sequence was 95% identical to the human sequence. Sequences were obtained from the NCBI GeneBank. [Human NM_030916.2; Orangutan(Pongo) XM_002809905.1; Cow NM_001024494.1; Dog XM_847277.1; Rat NM_001109076.1; Mouse NM_027893.3]. See also Figure 9.

Figure S7. Other wild type strains of MV (Montefiore 89 and WTF) and the Edmonston vaccine strain of MV can also use PVRL4 as a receptor. (A,B) Following transfection of the PVRL4 expression vector, OMK (owl monkey kidney) cells which lack a complete CD46 receptor, became susceptible to Edmonston vaccine MV. (C,D) HeLa cells transfected with the PVRL4 expression plasmid became susceptible to WTFH-EGFP MV infection. (E,F) HEK (293) cells transfected with PVRL4 become permissive to Montefiore 89 wtMV infections. In this case cells were fixed with paraformaldehye, permeabilized with 0.1% TX-100 detergent, and stained with measles (H, M) antibodies and detected with Alexa Fluor 488 conjugated goat anti-mouse secondary antibodies. Nuclei were stained with Hoechst stain. Scale bar=100µm. See also Figures 2, S1, and S2.

Table S1. Excel file showing normalized probe set intensity values and % up-regulation of membrane protein expression in MV permissive breast, lung, and primary SAEC. Text files from Expression Console were exported to Microsoft Excel. Control data and non-annotated entries were discarded. Data was filtered for membrane proteins by using the Excel filter function for the GO cellular component term “membrane”, and this data was retained. Intensity values for the non-permissive cells (MDA-MB-231 and A549) were averaged and compared to the averaged probe set values of permissive breast and lung arrays and expressed as a percentage up or down regulation (Sheets 1 and 2). Regulation of membrane protein gene products in serum

activated SAEC compared to SAEC grown in serum was calculated in a similar fashion (Sheet 3). Probe set gene entries for each of the tissue types (breast, lung, SAEC) were ranked in descending order (percentage up/down regulation - high to low). See also Table 2.

Supplemental Experimental Procedures

Microarray Analysis

Total RNA was extracted from SAEC and adenocarcinoma cell lines using the Qiagen RNeasy kit. The quality and quantity of RNA was assessed by both A260/A280 values and using an Agilent RNA BioAnalyzer. Microarray analysis was performed at the Applied Genomics Centre associated with the Toronto Hospital for Sick Children by Xiolin Wang. The Applied Genomics Centre is an accredited Affymetrix Service Provider. cDNA (5.5μg in 220μl) was transcribed and biotin end-labeled using the Affymetrix IVT kit. The fragmented probe was hybridized to the Human Gene 1.0 ST Array cartridge and washed using the FS450_0007 protocol, and stained with streptavidin-PE. The GeneChip was scanned with an Affymetrix GeneChip Scanner 3000. Chip data was analyzed by GCOS 1.4 and archived on DVD discs as GCOS DTT Files which included raw intensity CEL files and normalized CHP files. Microarray data was evaluated for Quality Control by the Applied Genomics Centre and transmitted to our laboratory. Data was further analyzed with Affymetrix Expression Console 1.1 software using the hugene-1_0st v.1 na30.hg19 annotation file. Normalized probe set intensity values on a scale of 0 (no signal) to 14 (strongest signal) were converted to a text file and exported to Microsoft Excel 2003 for further analysis. Negative and positive control data was discarded and the Probe Set's were filtered using the Excel filter function for the GO cellular component term "membrane", and this data was retained. Gene up-regulation was calculated by applying the formula [(SAEC with FCS) - (SAEC without FCS)] / (SAEC without FCS) to the normalized microarray intensity values and

expressing up-regulation as a percentage. Gene up-regulation in permissive vs. non-permissive cells lines was obtained by applying the formula [(permissive) - (non-permissive)] / (non-permissive) to the microarray intensity values and expressing up-regulation as a percentage. Average gene up-regulation was determined for breast cancer (MCF7, MDA-MB-468, and T47D), lung cancer (MGH-24, NCI-H125, and NCI-H358) and SAEC cell lines (Table S1). Membrane protein genes which were up-regulated >20% were compared between permissive breast cell lines and permissive lung cell lines, and then with serum activated permissive SAEC using the Excel function [=(ISERROR(MATCH(A1,C1:CN,0)),"",A1)], where A contains the Gene Names in the cell type (eg. Breast) compared to the Gene Names (C) in another cell type (eg. Lung). N represents the number of entries in the list being compared. Up-regulated gene products that were common between the different permissive cell lines are tabulated in Figure 2.

Immune histochemistry protocol for staining PVRL4 in human tissue sections

Formalin fixed paraffin embedded tissue was sliced at a 4 μm thickness with a microtome and dried in a 60°C oven overnight. Sections were dewaxed in xylene and rehydrated through graded concentrations of alcohol to water. Endogenous peroxidase was blocked with 3% hydrogen peroxide. Heat induced epitope retrieval in 10 mM citrate buffer, pH 6.0 was performed in a Milestone T/T Mega microwave oven. After blocking for endogenous biotin using Vector's biotin blocking kit, sections were incubated in primary antibody (anti-Nectin-4, R&D Systems, goat polyclonal, 1:1000 dilution) for 16 hours at room temperature in a humidified chamber. After washing the sections in PBS, secondary incubations were carried out with biotin-anti-goat IgG (Vector Laboratories), followed by incubation with streptavidin-HRP (ID Laboratories) for 30 min. Antibody binding was revealed by treating the sections with DAB substrate (Dako

North America, Inc) for 5 min. Samples were counterstained with Mayer's haematoxylin and mounted in Permount.

Figure S1

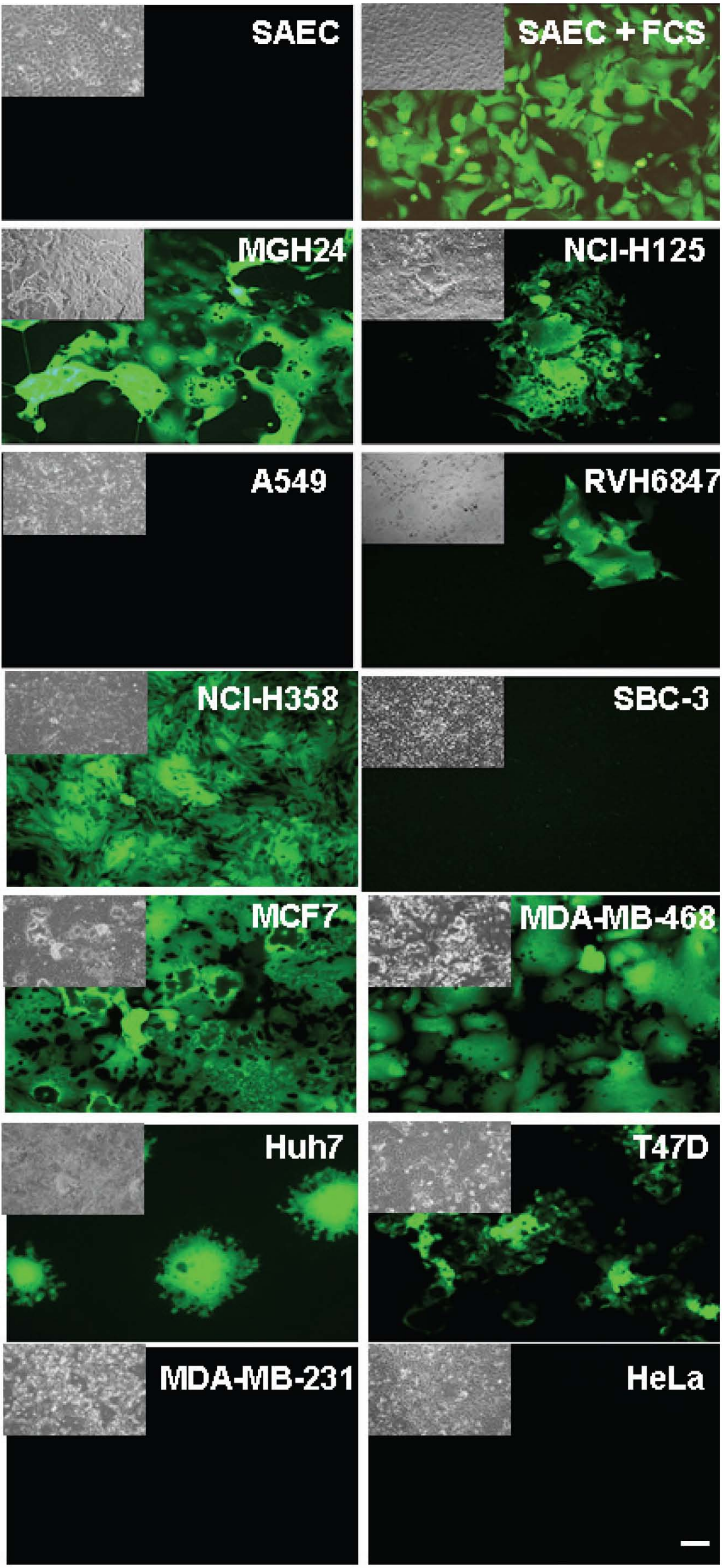
SAEC
SAEC + FCS
MGH24
NCI-H125
A549
RVH6847
NCI-H358
SBC-3
MCF7
MDA-MB-468
Huh7
T47D
MDA-MB-231
HeLa

Figure S2

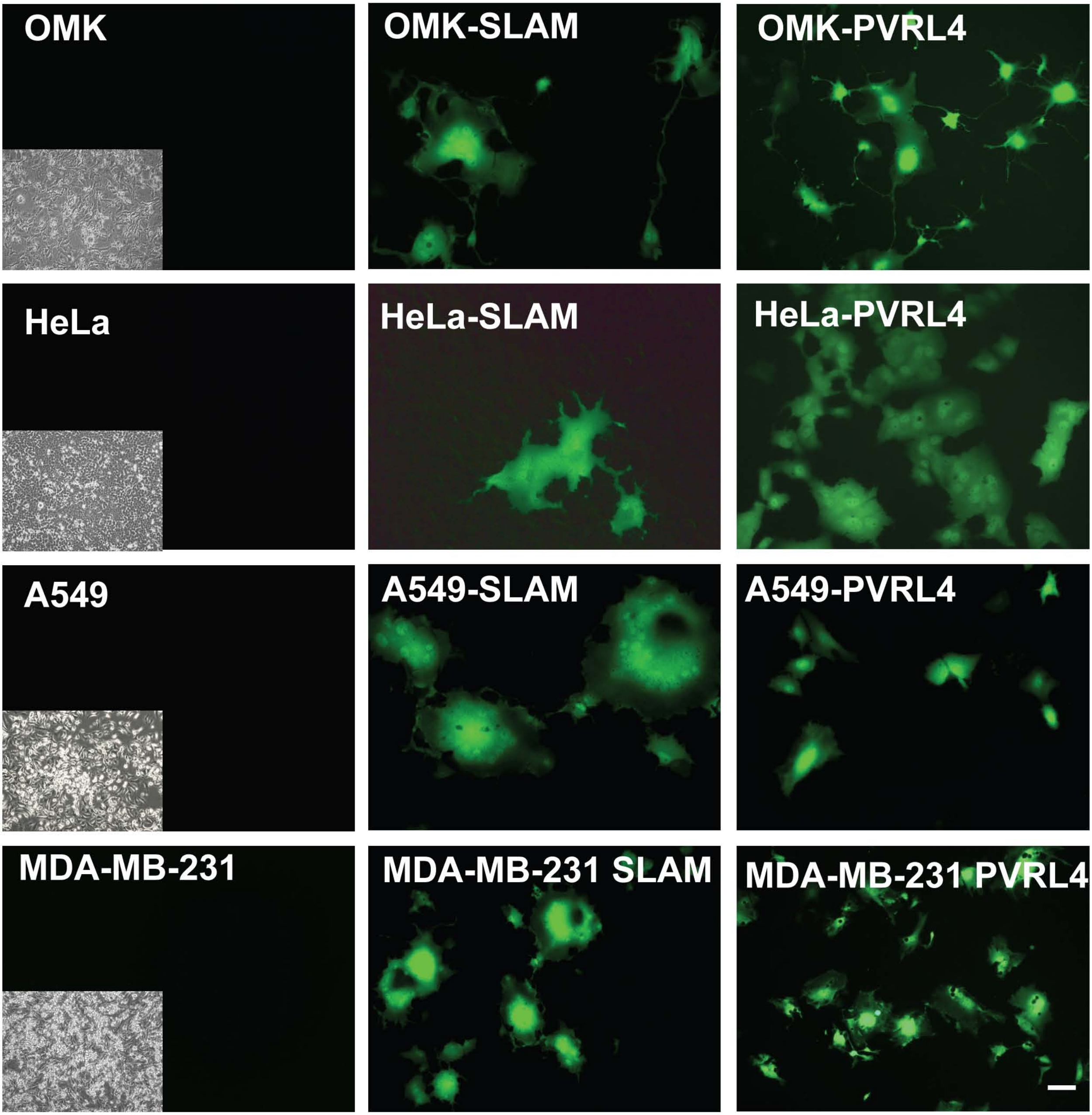
OMK
OMK-SLAM
OMK-PVRL4
HeLa
HeLa-SLAM
HeLa-PVRL4
A549
A549-SLAM
A549-PVRL4
MDA-MB-231
MDA-MB-231 SLAM
MDA-MB-231 PVRL4

Figure S3

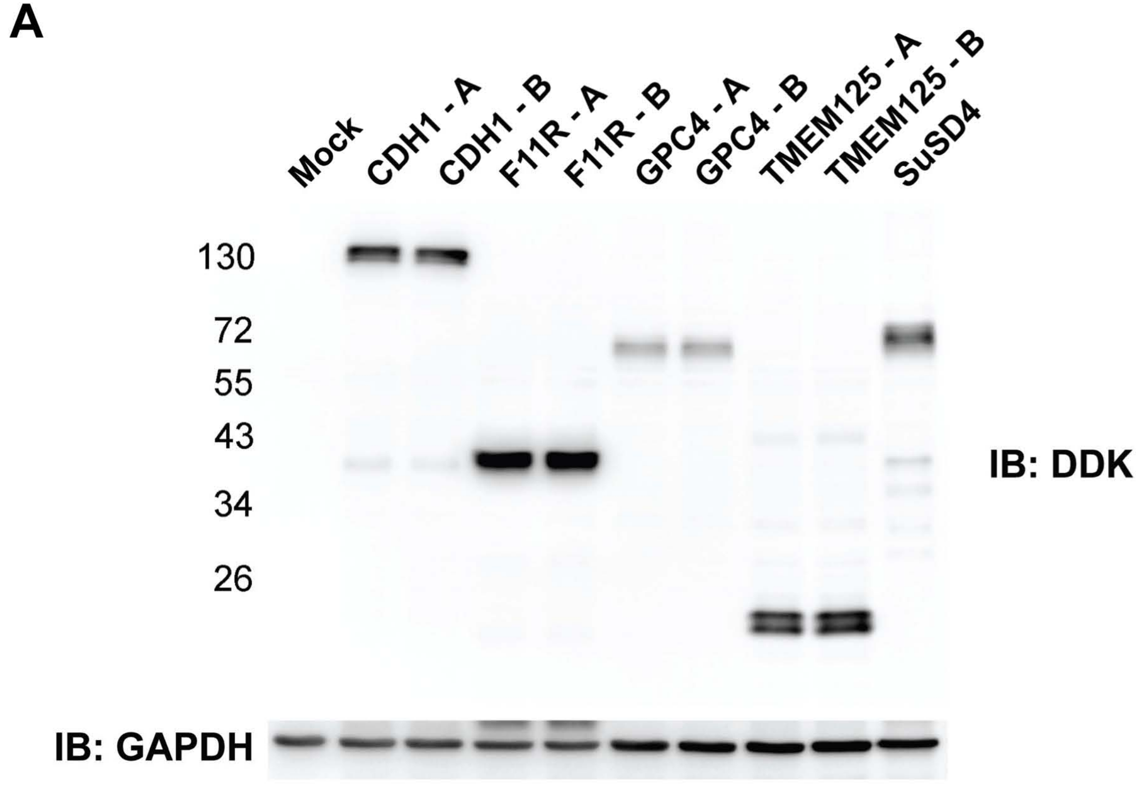
A
Mock
CDH1 - A
CDH1 - B
F11R - A
F11R - B
GPC4 - A
GPC4 - B
TMEM125 - A
TMEM125 - B
SuSD4
130
72
55
43
34
26
IB: DDK
IB: GAPDH

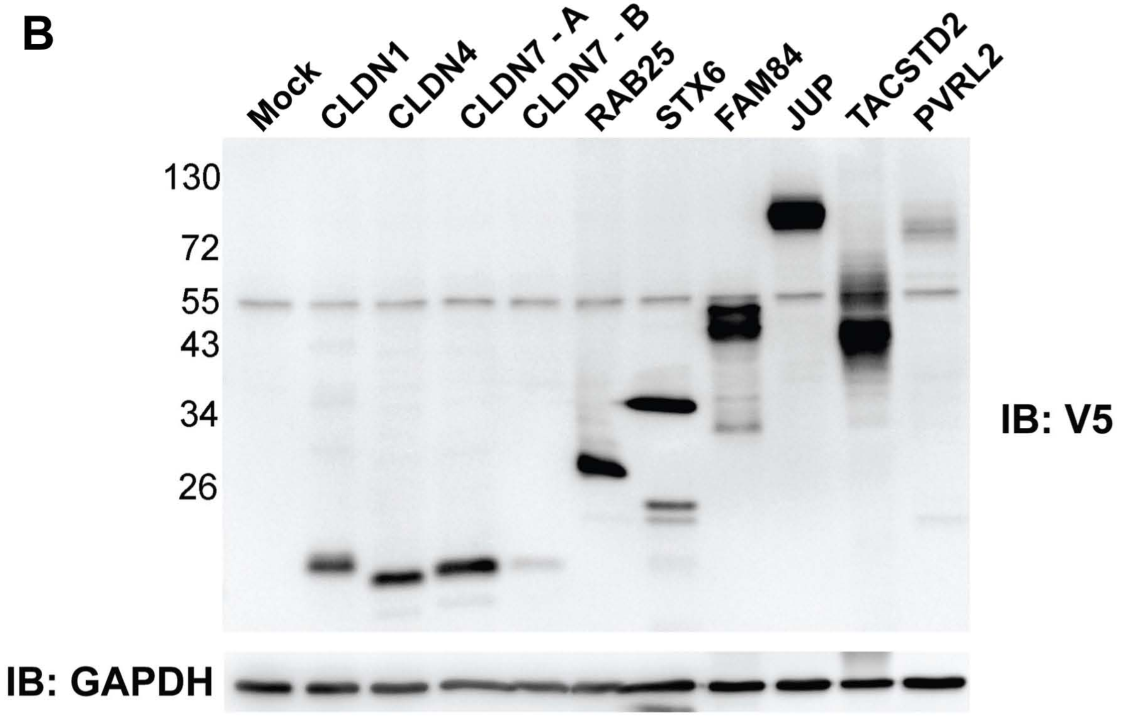
B
Mock
CLDN1
CLDN4
CLDN7 - A
CLDN7 - B
RAB25
STX6
FAM84
JUP
TACSTD2
PVRL2
130
72
55
43
34
26
IB: V5
IB: GAPDH

Figure S4

```
Human PVRL1.pro   MARM----GLAGAAGR-------WWGLALGLTA---------FF-------LPGVHSQVV   33
Human PVRL2.pro   MAR----------------------AAALLPSRSPPTPLLWPLLLLLLLET--GAQDVRV   36
Human PVRL3.pro   MARTLRPSPLCPGGGKAQLSSASLLGAGLLLQPPTPPPLLLLLFPLLLFSRLCGALAGPI   60
Human PVRL4.pro   MPLSL---------------GAEMWG-----------PEAWLLLLLLLASFTGRCPAGEL   34

Human PVRL1.pro   QVNDSMYGFIGTDVVLHCSFANPLPSVKITQVTWQKSTNGSK-QNVAIYNPSMG--VSVL   90
Human PVRL2.pro   QVLPEVRGQLGGTVELPCHLLPPVPGLYISLVTWQRPDAPANHQNVAAFHPKMGPSFPSP   96
Human PVRL3.pro   IVEPHVTAVWGKNVSLKCLIE---VNETITQISWEKIHGKSS-QTVAVHHPQYG--FSVQ  114
Human PVRL4.pro   ETSDVVTVVLGQDAKLPCFYRGD-SGEQVGQVAWARVDAGEGAQELALLHSKYG--LHVS   91

Human PVRL1.pro   APYRERVEFLRPS----------FTDGTIRLSRLELEDEGVYICEFATFPTGNRESQLNL  140
Human PVRL2.pro   KPGSERLSFVSAKQSTGQDTEAELQDATLALHGLTVEDEGNYTCEFATFPKGSVRGMTWL  156
Human PVRL3.pro   GEYQGRVLFKNYS----------LNDATITLHNIGFSDSGKYICKAVTFPLGNAQSSTTV  164
Human PVRL4.pro   PAYEGRVEQPPPPRNP-------LDGSVLLRNAVQADEGEYECRVSTFPAGSFQARLRL  143

Human PVRL1.pro   TVMAKPT-NWIEGTQAVLRAKKGQDDKVLVATCTSANGKPPSVVSWETRLKGEAEYQEIR  199
Human PVRL2.pro   RVIAKPK------NQAEAQKVTFSQDPTTVALCISKEGRPPARISWLSSLDWEAKETQVS  210
Human PVRL3.pro   TVLVEPTVSLIKGPDSLI----DGGNETVAAICIAATGKPVAHIDWEGDL-GEMESTTTS  219
Human PVRL4.pro   RVLVPPLPSLNPGP-----ALEEGQGLTLAASCTA-EGSPAPSVTWDTEVKGTTSSRSFK  197

Human PVRL1.pro   NP-NGTVTVISRYRLVPSREAHQQSLACIVNYH-MDR-FKESLTLNVQYEPEVTIEGFDG  256
Human PVRL2.pro   GTLAGTVTVTSRFTLVPSGRADGVTVTCKVEHESFEEPALIPVTLSVRYPPEVSISGYDD  270
Human PVRL3.pro   FP-NETATIISQYKLFPTRFARGRRITCVVKHPALEKDIRYSFILDIQYAPEVSVTGYDG  278
Human PVRL4.pro   H--SRSAAVTSEFHLVPSRSMNGQPLTCVVSHPGLLQDQRITHILHVSFLAEASVRGLED  255

Human PVRL1.pro   N--WYLQRMDVKLTCKADANPPATEYHWTTLNGSLPKGVEAQNRTLFFKGPINYSLAGTY  314
Human PVRL2.pro   N--WYLGRTDATLSCDVRSNPEPTGYDWSTTSGTFPTSAVAQGSQLVI-HAVDSLFNTTF  327
Human PVRL3.pro   N--WFVGRKGVNLKCNADANPPPFKSVWSRLDGQWPDGLLASDNTLHFVHPLTFNYSGVY  336
Human PVRL4.pro   QNLWHIGREGAMLKCLSEGQPPP-SYNWTRLDGPLPSGVRVDGDTLGF-PPLTTEHSGIY  313

Human PVRL1.pro   ICEATNPIGTRSGQVEVNITE---------------------------FPYTPSPPEH  345
Human PVRL2.pro   VCTVTNAVGMGRAEQVIFVRETPNTAGAGAT------GGIIGGIIAAIIATAVAATGILI  381
Human PVRL3.pro   ICKVTNSLGQRSDQKVIYISDPPTTTTLQPTIQWHPSTADIEDLATEPKKLPF-PLSTLA  395
Human PVRL4.pro   VCHVSNEFSSRDSQVTVDVLDPQEDSGKQ--VDLVSASVVVVGVIAALLFCLLVVVVVLM  371

Human PVRL1.pro   GRRAGPVPTAIIGGVAGSILLVLIVVGGIVVALRRRRHTFKGDYSTKKHVYGNGYSKAGI  405
Human PVRL2.pro   C-RQQRKEQTLQGAEEDEDLEGPPSYKPPTPKAKLEAQEMPSQLFTLGASEHSPLKTPYF  440
Human PVRL3.pro   TIKDDTIATIIASVVGGALFIVLVSVLAGIFCYRRRR-TFRGDYFAKNYIPPSDMQKE--  452
Human PVRL4.pro   SRYHRRKAQQMTQKYEEELTLTRENSIRRLHSHHTDPRSQPEESVGLRAEGHPDSLKDNS  431

Human PVRL1.pro   PQHHPPMAQNLQ-YPD-DSDDEKKA--GPLGGSSYEEEEEEEEGGGGGERKVGGPHPKYD  461
Human PVRL2.pro   DAGASCTEQEMPRYHELPTLEERSGPLHP--GATSLGSPIPVPPGPPAVEDVSLDLEDEE  498
Human PVRL3.pro   SQIDVLQQDELDSYPD-SVKKENKNPVNNLIRKDYLEEPEKTQ------WNNVENLNRFE  505
Human PVRL4.pro   SCSVMSEEPEGRSYSTLTTVREIETQTELLS-----------PGSG----------RAE  469

Human PVRL1.pro   EDAKRPYFTVDEAEARQDGYGDRTLGYQYDPEQL--DLAENMVSQNDGSFISKKEWYV  517
Human PVRL2.pro   GEEEEEYL-----DKINPIYDALSYS---SPSDSYQGKG----------FVMSRAMYV  538
Human PVRL3.pro   ----RP----------MDYYEDLKMGMKFVSDEHYDENEDDLVSHVDGSVISRREWYV  549
Human PVRL4.pro   EEEDQD---EGIKQAMNHFVQENGTLRAKPTG-------------NGIYINGRGHLV  510
```

Figure S5

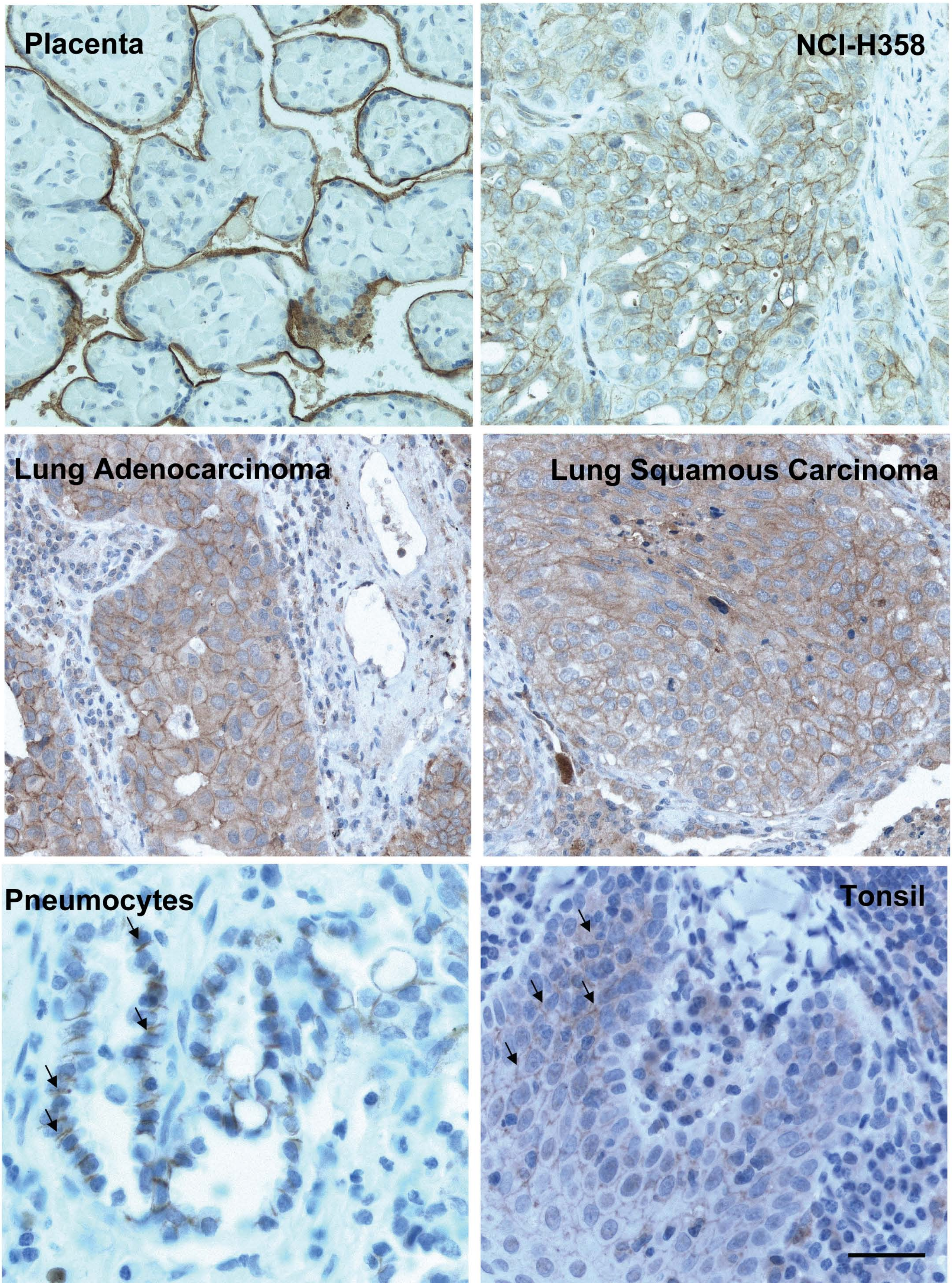
Placenta
NCI-H358
Lung Adenocarcinoma
Lung Squamous Carcinoma
Pneumocytes
Tonsil

Figure S6

```
Human PVRL4.pro    MPLSLGAEMWGPEAWLLLLLLLASFTGRCPAGELETSDVVTVVLGQDAKLPCFYRGDSGE  60
Pongo PVRL4.pro    MPLSLGAEMWGPEAWLLLLLLLASFTGRCRAGELETSDVVTVVLGQDAKLPCFYRGDSGE  60
Canine PVRL4.pro   MPLSLGAEMWGPEV-LLLLLLLASFTGRCRPGELETSDLVTVVLGQDAKLPCFYRGDPGE  59
Rat PVRL4.pro      MPLSLGAEMWGPEAWLLLLFL-ASFTGRYSAGELETSDLVTVVLGQDAKLPCFYRGDPDE  59
Mouse PVRL4.pro    MPLSLGAEMWGPEAWLRLLFL-ASFTGQYSAGELETSDVVTVVLGQDAKLPCFYRGDPDE  59

Human PVRL4.pro    QVGQVAWARVDAGEGAQELALLHSKYGLHVSPAYEGRVEQPPPPRNPLDGSVLLRNAVQA  120
Pongo PVRL4.pro    QVGQVAWARVDAGEGAQELALLHSKYGLHVSPAYEGRVEQPPPPRNPLDGSVLLRNAVQA  120
Canine PVRL4.pro   QVGQVAWARVDAGEGARELALLHSKYGLHVSAAYEGRVEQPPPPRSPLDGAVLLRNAVQA  119
Rat PVRL4.pro      QVGQVAWARVDPNEGTRELALLHSKYGLHVSPAYEDRVEQPPPPRDPLDGSILLRNAVQA  119
Mouse PVRL4.pro    QVGQVAWARVDPNEGIRELALLHSKYGLHVNPAYEDRVEQPPPPRDPLDGSVLLRNAVQA  119

Human PVRL4.pro    DEGEYECRVSTFPAGSFQARLRLRVLVPPLPSLNPGPALEEGQGLTLAASCTAEGSPAPS  180
Pongo PVRL4.pro    DEGEYECRVSTFPAGSFQARLRLRVLVPPLPSLNPGPALEEGQGLTLAASCTAEGSPAPS  180
Canine PVRL4.pro   DEGEYECRVSTFPAGSFQARLRLRVLVPPLPSLNPGPALEEGQGLTLAASCTAEGSPAPS  179
Rat PVRL4.pro      DEGEYECRVSTFPAGSFQARMRLRVLVPPLPSLNPGPPLEEGQGLTLAASCTAEGSPAPS  179
Mouse PVRL4.pro    DEGEYECRVSTFPAGSFQARMRLRVLVPPLPSLNPGPPLEEGQGLTLAASCTAEGSPAPS  179

Human PVRL4.pro    VTWDTEVKGTTSSRSFKHSRSAAVTSEFHLVPSRSMNGQPLTCVVSHPGLLQDQRITHIL  240
Pongo PVRL4.pro    VTWDTEVKGTTSSRSFKHSRSAAVTSEFHLVPSRSMNGQPLTCVVSHPGLLQDQRITHIL  240
Canine PVRL4.pro   VTWDTEVKGTASSRSFKHSRSAAVTSEFHLVPSRSMNGQPLTCVVSHPGLLQDQRITHVL  239
Rat PVRL4.pro      VTWDTEVKGTQSSRSFKHSRSAAVTSEFHLVPSRSMNGQPLTCVVSHPGLLQDQRITHTL  239
Mouse PVRL4.pro    VTWDTEVKGTQSSRSFTHPRSAAVTSEFHLVPSRSMNGQPLTCVVSHPGLLQDRRITHTL  239

Human PVRL4.pro    HVSFLAEASVRGLEDQNLWHIGREGAMLKCLSEGQPPPSYNWTRLDGPLPSGVRVDGDTL  300
Pongo PVRL4.pro    HVSFLAEASVRGLEDQNLWHVGREGAMLKCLSEGQPPPSYNWTRLDGPLPSGVQVDGDTL  300
Canine PVRL4.pro   QVAFLAEASVRGLEDQKLWQVGREGATLKCLSEGHPPPSYNWTRLDGPLPSGVRVQGDTL  299
Rat PVRL4.pro      QVAFLAEASVRGLEDQNLWHVGREGATLKCLSEGQPPPKYNWTRLDGPLPSGVRVKGDTL  299
Mouse PVRL4.pro    QVAFLAEASVRGLEDQNLWQVGREGATLKCLSEGQPPPKYNWTRLDGPLPSGVRVKGDTL  299

Human PVRL4.pro    GFPPLTTEHSGIYVCHVSNEFSSRDSQVTVDVLDPQEDSGKQVDLVSASVVVVGVIAALL  360
Pongo PVRL4.pro    GFPPLTTEHSGIYVCHVSNEFSSRDSQVTVDVLDPQEDSGKQVDLVSASVVVVGVIAALL  360
Canine PVRL4.pro   GFPPLTAEHSGTYVCHVSNELSSRDSQVTVDVLDPEEAPGKQVDLVSASVVVVGVIAALL  359
Rat PVRL4.pro      GFPPLTTEHSGVYVCHVSNELSSRASQVTVEVLDP-EDPGKQVDLVSASVVVVGVIAALL  358
Mouse PVRL4.pro    GFPPLTTEHSGVYVCHVSNELSSRDSQVTVEVLDP-EDPGKQVDLVSASVIIVGVIAALL  358

Human PVRL4.pro    FCLLVVVVVLMSRYHRRKAQQMTQKYEEELTLTRENSIRRLHSHHTDPRSQPEESVGLRA  420
Pongo PVRL4.pro    FCLLVVVVVLMSRYHRRKAQQMTQKYEEELTLTRENSIRRLHSHHTDPRSQPEESVGLRA  420
Canine PVRL4.pro   FCLLVVVVVLMSRYHRRKAQQMTQKYEEELTLTRENSIRRLHSHHSDPRSQPEESVGLRA  419
Rat PVRL4.pro      FCLLVVVVVLMSRYHRRKAQQMTQKYEEELTLTRENSIRRLHSHHTDPRSQPEESVGLRA  418
Mouse PVRL4.pro    FCLLVVVVVLMSRYHRRKAQQMTQKYEEELTLTRENSIRRLHSHHSDPRSQPEESVGLRA  418

Human PVRL4.pro    EGHPDSLKDNSSCSVMSEEPEGRSYSTLTTVREIETQTELLSPGSGRAEEEEDQDEGIKQ  480
Pongo PVRL4.pro    EGHPDSLKDNSSCSVMSEEPEGRSYSTLTTVREIETQTELLSPGSGRAEEE-DQDEGIKQ  479
Canine PVRL4.pro   EGHPDSLKDNSSCSVMSEEPEGRSYSTLTTVREIETQTELLSPGSGRAEEEEDRDEGIKQ  479
Rat PVRL4.pro      EGHPDSLKDNSSCSVMSEEPEGRSYSTLTTVREIETQTELLSPGSGRTEEEDDQDEGIKQ  478
Mouse PVRL4.pro    EGHPDSLKDNSSCSVMSEEPEGRSYSTLTTVREIETQTELLSPGSGRTEEDDDQDEGIKQ  478

Human PVRL4.pro    AMNHFVQENGTLRAKPTGNGIYINGRGHLV                                510
Pongo PVRL4.pro    AMNHFVQENGTLRAKPTGNGIYINGRGHLV                                509
Canine PVRL4.pro   AMNHFVQENGTLRAKPTGNGIYINGRGHLV                                509
Rat PVRL4.pro      AMNHFVQENGTLRAKPTGNGIYINGRGHLV                                508
Mouse PVRL4.pro    AMNHFVQENGTLRAKPTGNGIYINGRGHLV                                508
```

Figure S7

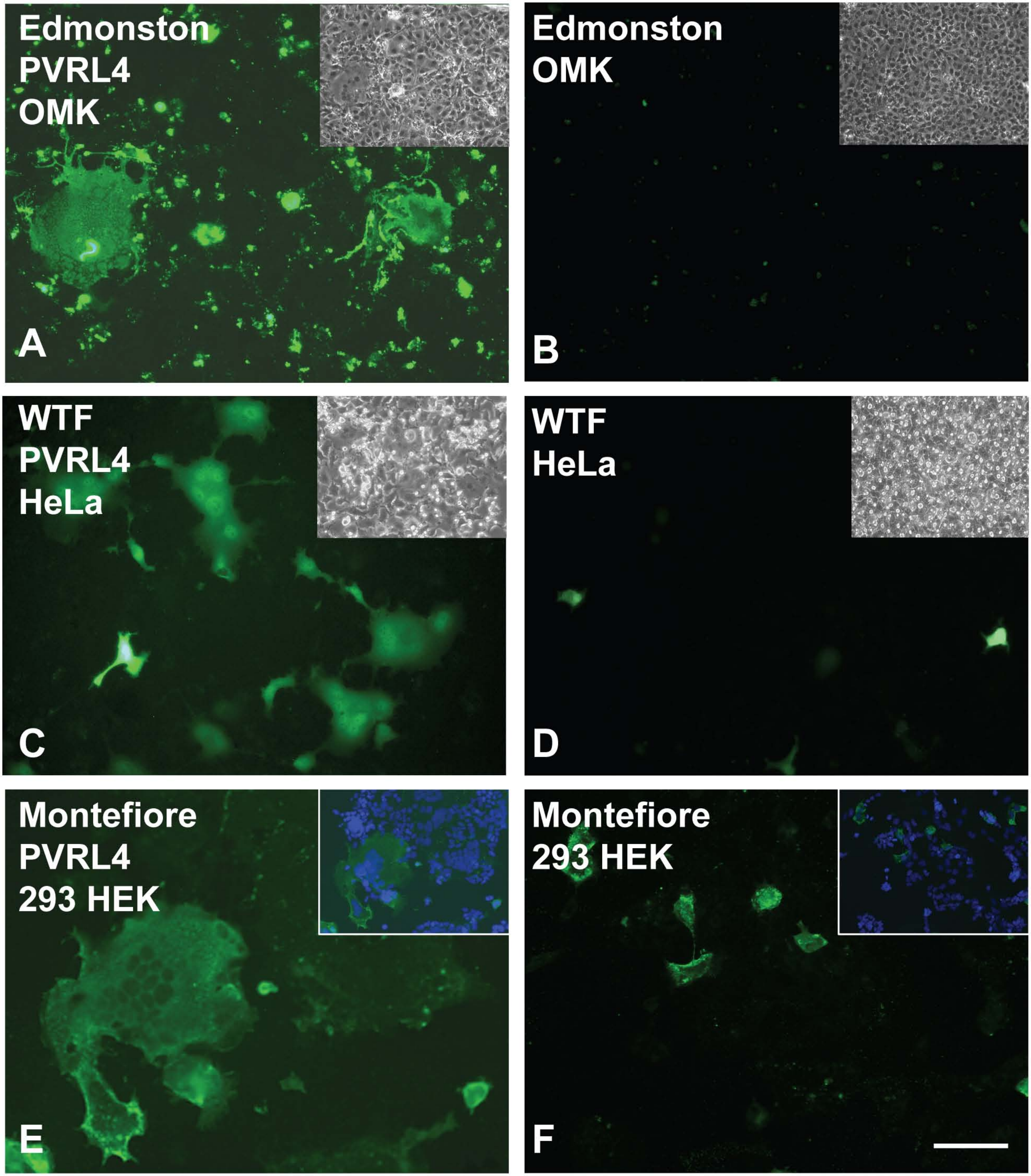

Edmonston
PVRL4
OMK
A
Edmonston
OMK
B
WTF
PVRL4
HeLa
C
WTF
HeLa
D
Montefiore
PVRL4
293 HEK
E
Montefiore
293 HEK
F
